# Correlates of Nucleocapsid Antibodies and a Combination of Spike and Nucleocapsid Antibodies Against Protection of SARS-CoV-2 Infection During the Omicron XBB.1.16/EG.5–Predominant Wave

**DOI:** 10.1093/ofid/ofae455

**Published:** 2024-08-28

**Authors:** Shohei Yamamoto, Yusuke Oshiro, Natsumi Inamura, Takashi Nemoto, Tomofumi Tan, Kumi Horii, Kaori Okudera, Maki Konishi, Tetsuya Mizoue, Haruhito Sugiyama, Nobuyoshi Aoyanagi, Wataru Sugiura, Norio Ohmagari

**Affiliations:** Department of Epidemiology and Prevention, Center for Clinical Sciences, National Center for Global Health and Medicine, Tokyo, Japan; Department of Laboratory Testing, Center Hospital of the National Center for the Global Health and Medicine, Tokyo, Japan; Department of Laboratory Testing, Center Hospital of the National Center for the Global Health and Medicine, Tokyo, Japan; Department of Laboratory Testing, Center Hospital of the National Center for the Global Health and Medicine, Tokyo, Japan; Department of Laboratory Testing, Center Hospital of the National Center for the Global Health and Medicine, Tokyo, Japan; Infection Control Office, Center Hospital of the National Center for the Global Health and Medicine, Tokyo, Japan; Infection Control Office, Kohnodai Hospital of the National Center for the Global Health and Medicine, Chiba, Japan; Department of Epidemiology and Prevention, Center for Clinical Sciences, National Center for Global Health and Medicine, Tokyo, Japan; Department of Epidemiology and Prevention, Center for Clinical Sciences, National Center for Global Health and Medicine, Tokyo, Japan; Center Hospital of the National Center for the Global Health and Medicine, Tokyo, Japan; Kohnodai Hospital of the National Center for the Global Health and Medicine, Chiba, Japan; Center for Clinical Sciences, National Center for Global Health and Medicine, Tokyo, Japan; Disease Control and Prevention Center, National Center for Global Health and Medicine, Tokyo, Japan

**Keywords:** COVID-19, nucleocapsid antibody, protection, reinfection, spike antibody

## Abstract

**Background:**

We aimed to examine the association among nucleocapsid (N) antibodies, a combination of N and spike (S) antibodies, and protection against SARS-CoV-2 reinfection.

**Methods:**

We conducted a prospective cohort study among staff at a national medical research center in Tokyo and followed them for the incidence of SARS-CoV-2 infection between June and September 2023 (Omicron XBB.1.16/EG.5 wave). At baseline, participants donated blood samples to measure N- and S-specific antibodies. Cox regression was used to estimate the hazard ratio and protection ([1 – hazard ratio] × 100) against subsequent SARS-CoV-2 infection across these antibody levels.

**Results:**

Among participants with previous infection, higher pre-reinfection N antibodies were associated with a lower risk of reinfection, even after adjusting S antibody levels (*P* < .01 for trend). Estimation of the protection matrix for N and S antibodies revealed that high levels in N and S antibodies conferred robust protection (>90%) against subsequent infection. In addition, a pattern of low pre-reinfection N antibodies but high vaccine-enhanced S antibodies showed high protection (>80%).

**Conclusions:**

Pre-reinfection N antibody levels correlated with protection against reinfection, independent of S antibodies. If the N antibodies were low, vaccine-boosted S antibodies might enhance the reinfection protection.

## Teaser

Pre-reinfection nucleocapsid antibody titers correlated with protection against SARS-CoV-2 reinfection during the Omicron XBB1.16/EG.5–predominant wave. High titers in nucleocapsid and spike conferred robust protection (>90%). If nucleocapsid antibodies were low, vaccine-boosted spike antibodies enhanced the reinfection protection (>80%).

Four years into the COVID-19 pandemic, >774 million COVID-19 cases have been reported worldwide as of January 2024 [1]. Currently, reinfection with the persistently emerging Omicron variants has become an important public health concern, underscoring the need to identify predictors for prevention. Previous SARS-CoV-2 infection is associated with a lower risk of reinfection [2]. Among antigen-specific immune responses elicited during SARS-CoV-2, spike (S) protein–binding antibodies are known to have a role in preventing virus entry, and epidemiologic studies confirmed that higher S antibodies correlated with higher protection against reinfection [3, 4]. In contrast, the protective role of other antigen-specific immune responses remains unclear.

Nucleocapsid (N)–specific antibodies have been used as a marker of previous infection. Interestingly, in vivo studies showed that N antibodies elicited antibody-dependent cellular cytotoxicity effects [5] and were correlated with protection against the SARS-CoV-2 challenge [5–7]. Data from human epidemiologic studies are scarce and inconsistent. In a study among children with a history of COVID-19, higher N titers were associated with a significantly lower risk of reinfection during the Omicron BA.4/5 phase (2022) [8]. In a study of male adults, higher N titers were marginally associated with a lower risk of reinfection during the Delta-predominant wave (2021) [9]. In contrast, 2 other studies among adults with a small sample size (<120 individuals) reported no association between initial infection-acquired N antibody levels and risk of reinfection during the early pandemic phase (2020) [3, 10].

Besides the paucity of epidemiologic data linking N antibody levels to reinfection risk, some issues remain to be addressed. First, no studies controlled for S antibody levels when analyzing the association between N titer and reinfection risk. Because S and N antibodies are induced by infection and thus highly correlated, the lack of adjustment of S antibodies is critical when assessing the independent role of N antibodies. Second, no evidence is available in adults during the surge of the Omicron variants, wherein many infections and reinfections have occurred. Third, no studies investigated the combination of S and N antibody levels in relation to the risk of reinfection. In vivo studies showed that mice with S and N antibodies had better protection than those with S antibodies only [11, 12], suggesting that higher levels in S and N antibodies may confer robust protection.

Here, we studied the association of pre-reinfection N antibody level and a combination of S and N antibodies with the risk of SARS-CoV-2 reinfection during the Omicron XBB.1.16/EG.5–predominant wave among the staff of a national medical and research center in Tokyo.

## METHODS

### Study Setting

In the National Center for Global Health and Medicine (NCGM) in Japan, a repeat serologic study was launched in July 2020 to monitor the spread of SARS-CoV-2 infection among staff during the COVID-19 epidemic. The details of this study have been reported elsewhere [13, 14]. In summary, we have completed 8 serosurveys as of June 2023. We measured anti-SARS-CoV-2 N-protein antibodies (all serosurveys) and S-protein antibodies (from the second serosurvey onward) for all participants, using Roche and Abbott assays and stored serum samples at −80 °C. We also measured N and S antibodies with Sysmex assays in 4 serosurveys (once a year). In addition, we collected information on COVID-19–related factors via a questionnaire, including vaccination, occupational infection risk, and infection prevention practices. Self-reported vaccination status was validated against the vaccine records of the NCGM Labor Office. Written informed consent was obtained from all participants. This study was approved by the NCGM Ethics Committee (NCGM-G-003598).

### Analytic Cohort

In the present prospective study, we set the baseline cohort as all participants who attended the eighth survey, conducted in June 2023, where we invited all NCGM staff (n = 3206) to participate and 2569 (80%) completed a questionnaire and donated blood samples. Of those, we excluded 20 participants who lacked information on covariates: body composition (n = 12), alcohol-drinking status (n = 3), living arrangement status (n = 6), adherence to infection prevention practice (n = 5), and infection risk behaviors (n = 3); thus, 2549 participants were analyzed.

### Ascertainment of Subsequent SARS-CoV-2 Infection

To assess the influence of Omicron XBB.1.16/EG.5.1, we followed the participants for COVID-19 incidence from baseline (June 2023) to September 2023, when these variants were predominant in Japan. SARS-CoV-2 infections were identified by COVID-19 patient records documented by the NCGM Hospital Infection Prevention and Control Unit, which provided information on the date of diagnosis, diagnostic procedures, symptoms, and hospitalizations. Per the NCGM rule, staff should undergo a polymerase chain reaction (PCR) or antigen test for COVID-19 when they have COVID-19–compatible symptoms, and if they test positive, they must report the results to the NCGM Hospital Infection Prevention and Control Unit. Some asymptomatic cases were also included in the registry if they tested positive (PCR or antigen test) for COVID-19 when they had close contact with a person with COVID-19 or when the staff who work in the same department were infected with COVID-19. Most registered cases were laboratory confirmed (PCR or antigen test), but there were a few exceptions, including those diagnosed by a physician with nonlaboratory information (ie, symptoms compatible with COVID-19 after close contact with a patient with COVID-19).

In this analysis, we defined reinfection as being diagnosed >90 days after a previous diagnosis. However, no cases were rediagnosed within 90 days after the previous infection. We also considered the following pattern as reinfection: individuals who were first diagnosed with COVID-19 during follow-up but had a history of seropositive N antibodies at baseline.

### Antibody Testing

We assessed anti-SARS-CoV-2 N- and S-protein antibodies in all participants. N-specific antibodies was measured with 3 commercially available automated immunoassays: Elecsys Anti-SARS-CoV-2 (Roche Diagnostics), ARCHITECT SARS-CoV-2 IgG (Abbott Laboratories), and HISCL SARS-CoV-2 N-IgG (Sysmex Co) [15]. The antibodies measured with Roche (cutoff index) and Abbott (signal to cutoff) are qualitative, while those with Sysmex (Sysmex unit: SU/mL) are semiquantitative (ie, quantitative results but are not calibrated to the World Health Organization standard). Abbott and Sysmex assays measure immunoglobulin G (IgG) N, whereas the Roche assay measures total N, including IgG. We also quantitatively measured the antibodies against the receptor-binding domain (RBD) of the SARS-CoV-2 S protein using the Elecsys Anti-SARS-CoV-2 S (Roche Diagnostics; ie, anti-RBD total, U/mL) and the AdviseDx SARS-CoV-2 IgG II assay (Abbott Laboratories; ie, anti-RBD IgG, AU/mL) and that against the SARS-CoV-2 IgG S protein using HISCL SARS-CoV-2 S-IgG (Sysmex Co; ie, anti-S IgG, BAU/mL). The Sysmex IgG-N and IgG-S assays were available only in Japan, and the package insert of these assays (in Japanese) is attached as Supplementary Documents 1 and 2.

### Previous SARS-CoV-2 Infection Status at Baseline

Previous infection was defined as (1) a history of COVID-19 confirmed against an in-house COVID-19 registry at baseline, (2) a history of self-reported COVID-19 via a questionnaire, or (3) anti-N seropositivity with any of the 3 assays (Roche, ≥1.0 cutoff index; Abbott, ≥1.40 signal to cutoff; or Sysmex, ≥10 SU/mL) at any of the first through eighth surveys (July 2020 to June 2023 [baseline]). Since the in-house registry did not record the COVID-19 history of those infected before they began working at the NCGM, we also used information on the self-reported COVID-19 history. Participants were dichotomized by infection status at baseline: infection naive or previously infected. The latter was divided into quartile groups according to the N antibody level on each of the 3 assays.

### Statistical Analysis

We calculated the person-time from the date of the baseline blood sampling (13–23 June 2023) to the date of subsequent SARS-CoV-2 infection, receiving an additional COVID-19 vaccine, or censoring (6 September 2023), whichever occurred first. If participants received an additional COVID-19 vaccine, follow-up was censored at 13 days postvaccination, assuming that they were not sufficiently immunized with the additional booster until then. We fitted a Cox proportional hazard regression analysis to examine the association between N antibody levels (ie, infection-naive group and N index quartile groups of those previously infected) and the risk of subsequent SARS-CoV-2 infection during the Omicron BBX.1.16/EG.5–predominant wave for each of the N antibodies measured via the 3 companies. Models were adjusted in the following manner. First, to control basic demographic factors, we adjusted for age and sex (model 1). Second, for the confounders of antibody titers and risk of infection, we additionally adjusted for job, occupational SARS-CoV-2 exposure risk, body mass index, comorbid diseases, immunosuppression, use of tobacco products, frequency of alcohol drinking, number of household members, infection prevention practice score, frequency of spending ≥30 minutes without a mask in the 3Cs (crowded places, close-contact settings, and confined and enclosed spaces), and frequency of having dinner in a group of ≥5 people for >1 hour (model 2). In the analyses of the association between N antibody titers and reinfection risk, we further adjusted the anti-S/RBD titer measured with the same company assay to investigate the protective role of N antibodies independent of the anti-S/RBD antibodies (model 3). To examine whether increased levels of the N index are associated with the decreased hazard ratios (HRs) among individuals with previous infection, we calculated the *P* value for the trend by treating the N index quartile variable as a continuous term ordinally coded 1, 2, 3, and 4 in the Cox models. We assessed the effect of multicollinearity for the multivariable models using the variance influence factor, and no significant effect was observed (variance influence factor ≤2).

Among the individuals previously infected, we examined the association between the N antibody index and the risk of SARS-CoV-2 reinfection using restricted cubic splines with 3 knots at the 10th, 50th, and 90th percentiles [16] of the N antibody distribution, which was based on the Cox proportional regression analysis with adjustment for the covariates of model 2. The *P* value for linearity was calculated by including a linear term of the N antibody index in the Cox model. The *P* value for non-linearity was also calculated by a likelihood ratio test comparing the model with only a linear term against the model with linear and cubic spline terms [17].

To examine the association between a combination of S and N antibody levels and protection against SARS-CoV-2 infection, we repeated the Cox regression model with adjustment for covariates of model 2. We used combined variables for each category of N antibody levels (ie, infection-naive group and N index quartile groups of those previously infected) and S antibody levels (ie, quartile groups) and set the reference group as infection naive and the lowest quartile of S antibodies. Furthermore, we fitted the Cox model while accounting for continuous S and log-N antibody titers as an interaction term, and the results of this analysis were conveyed visually in contour plots. In this contour plots model, the minimum values of S and N antibody titers were selected as reference values. The estimated HR was used to calculate protection (percentage) according to the following formula: (1 – HR) × 100.

For a sensitivity analysis, we repeated the previous analyses by restricting the outcome to symptomatic infection. For another sensitivity analysis, we ran the analyses after excluding nonregular staff (ie, contractors, temporary staff, café staff, shop staff, and part-time registered medical doctors) since the infection for nonregular staff might not be completely reported to the NCGM registry. Statistical analyses were performed with Stata version 18.0 (StataCorp LLC). All *P* values were 2-sided, and *P* < .05 was considered statistically significant.

## RESULTS

### Baseline Characteristics

Of 2549 participants, 70.9% were female, and the median age was 38 years (Table 1). The most frequent jobs were nurses (35.9%), followed by doctors (16.0%), allied health care workers (15.4%), administrative staff (15.2%), and researchers (12.2%). More than half (56%) showed some evidence of previous infection with SARS-CoV-2, whether registry-confirmed COVID-19 (53.2%), self-reported COVID-19 (15.1%), or seropositive with N antibodies (97%; Supplementary Figure 1). There was evidence that 54.6% of participants were seropositive with N antibodies on the Roche, Abbott, or Sysmex assay in the serosurveys in which they participated (ie, out of the 8 serosurveys from July 2020 to June 2023). Among participants, 50.0%, 13.5%, and 33.0% had evidence of N seropositivity on the Roche, Abbott, and Sysmex assays, respectively, in their serosurveys. Baseline characteristics stratified by previous infection status and Roche N index are summarized in Table 1. Individuals who were previously infected tended to be younger and at higher risk of occupational exposure to SARS-CoV-2, have fewer comorbidities, drink alcohol more frequently, engage in high-risk behaviors, live with more school-aged children, receive fewer doses of the COVID-19 vaccine, and have less frequent Omicron bivalent vaccination (BA.1 or BA.4/5) as compared with individuals who were infection naive. Among those with previous infection, a higher Roche N index was correlated with a shorter interval from the last COVID-19 diagnosis to the baseline and a higher number of COVID-19 diagnoses.

**Table 1.** Baseline Characteristics According to Anti-SARS-CoV-2 Nucleocapsid Index

			Roche Nucleocapsid Index Among Individuals Previously Infected			
Characteristics	Total	Infection Naive	Q1 (Lowest)	Q2	Q3	Q4 (Highest)
No. of participants	2549	1119	358	357	358	357
Female	70.9	73.0	70.4	68.3	69.3	68.6
Age, y	38 (27–49)	41 (29–53)	38 (28–47)	33 (26–45)	34 (27–46)	35 (27–48)
Job						
Doctor	16.0	11.6	22.1	18.2	17.0	20.4
Nurse	35.9	33.1	40.2	40.6	36.6	34.7
Allied health care worker^a^	15.4	17.9	10.3	14.6	15.9	13.2
Researcher	12.2	13.5	10.3	9.0	12.8	12.3
Administrative staff	15.2	18.4	12.0	11.5	12.3	14.8
Others	5.4	5.5	5.0	6.2	5.3	4.5
Occupational SARS-CoV-2 exposure risk^b^						
Low	61.2	65.1	60.1	56.0	60.3	56.3
Moderate	20.7	19.7	18.4	23.2	20.9	23.0
High	18.1	15.1	21.5	20.7	18.7	20.7
Body mass index, kg/m^2^	21.2 (19.5–23.4)	21.1 (19.4–23.6)	21.2 (19.7–23.3)	21.1 (19.4–23.0)	21.2 (19.5–22.9)	21.3 (19.7–23.5)
Comorbid diseases^c^	8.3	10.5	8.7	4.8	6.4	6.7
Immunosuppression^d^	1.0	1.0	0.8	0.6	1.7	0.8
Use of tobacco products^e^	7.6	8.3	5.6	8.4	7.0	7.3
Frequency of alcohol drinking						
None	32.9	37.2	30.7	28.6	31.6	27.5
Occasional	27.1	25.8	26.3	28.3	27.7	30.0
Weekly/daily	40.0	37.0	43.0	43.1	40.8	42.6
No. of household members	2 (1–3)	2 (1–3)	2 (1–4)	2 (1–4)	2 (1–4)	2 (1–4)
No. of school-aged children cohabiting^f^						
0	71.0	77.3	59.5	70.6	64.5	69.5
1	13.0	12.4	14.0	12.0	14.5	13.4
≥2	16.0	10.3	26.5	17.4	20.9	17.1
Infection prevention practice score^g^	7 (6–9)	7 (6–9)	7 (5–8)	7 (5–8)	7 (6–8)	7 (6–8)
Spending ≥30 min in the 3Cs without mask^h^						
None	62.0	67.6	54.2	58.8	60.3	57.1
1–5 times	29.1	27.5	33.5	29.4	29.1	29.4
≥6 times	8.9	4.8	12.3	11.8	10.6	13.4
Having dinner in a group of ≥5 people for >1 h^h^						
None	58.6	64.8	50.8	56.3	53.9	53.8
1–5 times	37.2	32.8	42.7	39.2	42.2	38.4
≥6 times	4.2	2.4	6.4	4.5	3.9	7.8
No. of previous COVID-19 diagnosis						
Never	60.1	100.0	33.2	30.5	22.6	29.4
1	38.4	0	65.9	69.5	75.4	63.0
2	1.4	0	0.8	0.0	1.7	7.3
3	0.1	0	0.0	0.0	0.3	0.3
Interval from last diagnosis to baseline, d	279 (183–342)	NA	332 (280–463)	309 (206–354)	239 (178–317)	190 (112–294)
Vaccination status, dose						
≤2	4.3	4.0	4.5	4.2	5.3	4.2
3	21.6	14.9	24.6	26.3	30.2	26.3
4	41.8	39.1	43.9	46.2	41.1	44.5
≥5	32.2	41.9	27.1	23.2	23.5	24.9
Receiver of Omicron bivalent vaccine	47.2	55.4	46.4	42.3	38.0	36.1
Interval from last vaccination to baseline, d	255 (181–306)	214 (178–299)	275 (182–457)	286 (182–455)	294 (186–461)	293 (186–452)

Data are presented as median (IQR) for continuous variables and percentage for categorical variables.

Abbreviations: 3Cs, crowded places, close-contact settings, and confined and enclosed spaces; NA, not applicable; Q, quartile.

^a^Allied health care workers include all health care professionals except for doctors and nurses (eg, pharmacists, laboratory/radiologic technologists, physical therapists/occupational therapists, and social workers).

^b^Occupational SARS-CoV-2 exposure risk was categorized as follows: low (those not engaged in COVID-19–related work), moderate (those engaged in COVID-19–related work without heavy exposure to SARS-CoV-2), or high (those heavily exposed to SARS-CoV-2). Details are described elsewhere [18].

^c^Comorbid diseases were defined as cancer, cardiovascular disease, diabetes, hypertension, chronic kidney disease, or lung disease.

^d^Immunosuppression was defined as having an immunosuppressive disease or using steroids (except topical or inhaled), immunosuppressants, or anticancer drugs.

^e^Tobacco products include conventional cigarettes and heated tobacco products.

^f^School-age children include those in nurseries, kindergartens, elementary to high school, and university and with disabilities.

^g^Calculation of infection prevention practice score was based on the total score of adherences to avoiding the 3Cs, hand washing, wearing a mask, and social distancing, as well as not touching the face, nose, or mouth, by assigning 2 points to “always,” 1 point to “often,” and 0 points to others (“seldom” and “not at all”).

^h^The self-reported number of times that the risk behavior was performed from March 2023 to June 2023 (baseline).

### Incidence of Subsequent SARS-CoV-2 Infection

During the follow-up, we identified 237 participants with SARS-CoV-2 infections, with an incident rate of 13.5 per 10 000 person-days. Of those, 192 cases were diagnosed for the first time, 42 for the second time, and 3 for the third time. The median interval between the diagnosed date of previous infection and that of reinfection was 388 days (range, 227–973). Among those diagnosed for the first time, 17 had a history of seropositivity on N antibodies at baseline, indicating that they had been infected at least 2 times. Most cases were symptomatic (95%), but all cases were not severe (ie, not hospitalized).

### Previous Infection Status, Pre-reinfection N Antibodies, and Subsequent SARS-CoV-2 Infection

**Figure 1.**
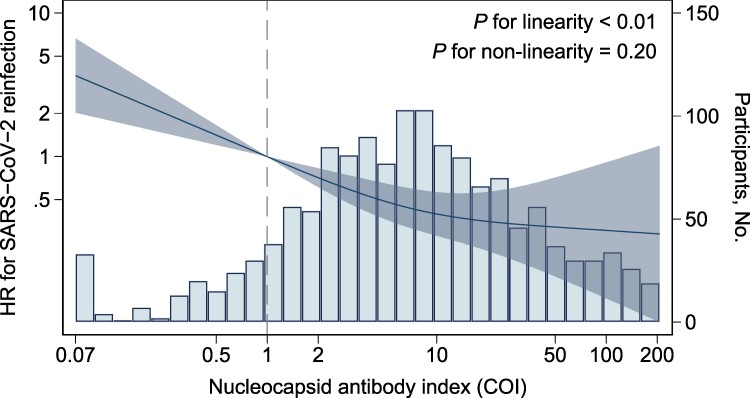
Association between anti-SARS-CoV-2 nucleocapsid antibody level and risk of reinfection among individuals previously infected. Solid lines indicate the hazard ratio for SARS-CoV-2 reinfection, and the shaded area represents 95% CIs. The bars indicate histograms of log-transformed nucleocapsid antibody levels. Reference points are seropositive thresholds for the Roche assay (1.0 COI). The model was adjusted for covariates of model 2 in Table 2. COI, cutoff index; HR, hazard ratio.

The infection-naive group had a higher risk of SARS-CoV-2 infection as compared with the previously infected group with the lowest quartile of the Roche N index, with an HR (95% CI) of 1.93 (1.32–2.83) in model 2 (Table 2). Within the previously infected groups, a higher pre-reinfection Roche N index was associated with a lower risk of reinfection: the HRs (95% CI) from the lowest to highest quartile groups were 1.00 (reference), 0.29 (.14–.57), 0.31 (.16–.60), and 0.14 (.05–.35), respectively, in model 2 (*P* < .01 for trend). The association was still significant after adjusting for the anti-RBD titer in model 3. The cubic spline analysis yielded a similar dose-response curve showing a steady decrease in HRs with a higher pre-reinfection Roche N index (*P* < .01 for linearity, *P* = .20 for non-linearity; Figure 1). These results were similar in the Abbott and Sysmex assays (Supplementary Table 1 and Supplementary Figure 2).

**Table 2.** Subsequent SARS-CoV-2 Infection Across the Baseline Anti-nucleocapsid Antibody Index

		Nucleocapsid Antibodies Among Individuals Previously Infected				
Roche Assay	Infection Naive	Q1 (Lowest)	Q2	Q3	Q4 (Highest)	*P* Value^a^
COI, median (range)	0.08 (0.07–0.98)	1.16 (0.07–2.46)	4.30 (2.48–7.14)	11.3 (7.15–20.7)	48.9 (20.8–256)	…
Cases/person-days, %^b^	175/71 901 (24.3)	34/24 958 (13.6)	11/25 897 (4.2)	12/26 564 (4.5)	5/26 677 (1.9)	…
Hazard ratio (95% CI)						
Model 1^c^	1.91 (1.32–2.77)	1 [Reference]	0.29 (.15–.58)	0.32 (.16–.61)	0.13 (.05–.34)	<.01
Model 2^d^	1.94 (1.33–2.83)	1 [Reference]	0.29 (.15–.57)	0.31 (.16–.60)	0.14 (.05–.35)	<.01
Model 3^e^	1.57 (1.06–2.32)	1 [Reference]	0.29 (.15–.58)	0.36 (.18–.69)	0.17 (.06–.42)	<.01

Abbreviations: 3Cs, crowded places, close-contact settings, and confined and enclosed spaces; COI, cutoff index; Q, quartile; RBD, receptor-binding domain.

^a^For trend among individuals previously infected.

^b^Incident rate per 10 000 person-days.

^c^Model 1 was adjusted for age (continuous) and sex (male or female).

^d^Model 2 was additionally adjusted for job (doctors, nurses, allied health professionals, researchers, administrative staff, or others), occupational SARS-CoV-2 exposure risk (low, moderate, or high), body mass index (continuous), comorbid diseases (no or yes), immunosuppression (no or yes), use of tobacco products (no or yes), frequency of alcohol drinking (none, occasional, or weekly/daily drinker), number of household members (continuous), infection prevention score (continuous), spending ≥30 minutes in the 3Cs without mask (none, 1–5 times, or ≥6 times), and having dinner in a group of ≥5 people for >1 hour (none, 1–5 times, or ≥6 times).

^e^Model 3 was further adjusted for anti-RBD titers measured with the Roche assay.

### Correlations Between N and S Antibodies

Low correlations were observed between N and S/RBD antibody levels among individuals who were previously infected: Spearman ρ (95% CI) between N and S/RBD antibodies as measured with the Roche, Abbott, and Sysmex assays was 0.23 (.18–.28), 0.32 (.28–.37), and 0.34 (.29–.38), respectively (Supplementary Figure 3). Irrespective of the N antibody levels, participants with a higher number of vaccine doses or shorter intervals from the last vaccination had higher levels of anti-S/RBD antibodies (Supplementary Table 2).

### A Joint Association of N and S Antibodies With Protection Against Subsequent SARS-CoV-2 Infection

**Figure 2.**
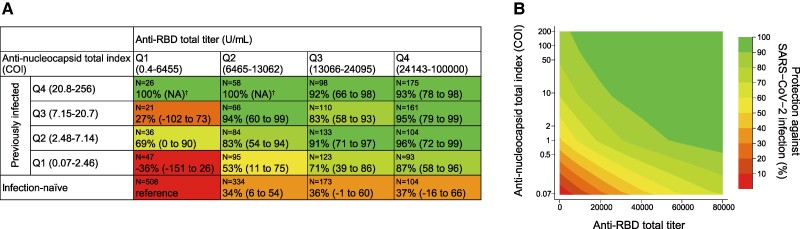
Infection protection matrices for the baseline anti-nucleocapsid and anti-RBD antibody levels. *A*, Matrix table of the protection against infection by categorical anti-nucleocapsid and anti-RBD antibodies, relative to the reference group of infection-naive antibodies and the lowest quartile of anti-RBD antibodies with the Roche assay. *B*, Also shown are contour plots of the protection by continuous anti-nucleocapsid and anti-RBD antibodies relative to reference values of the lowest value of anti-nucleocapsid and anti-RBD antibodies with Roche assays. Protection was calculated as (1 – hazard ratio) × 100. The hazard ratio was estimated by a Cox proportional hazards regression model, adjusting covariates of model 2 in Table 2. ^†^No incidence of subsequent SARS-CoV-2 infection in the group. COI, cutoff index; Q, quartile; NA, not applicable; RBD, receptor-binding domain.

Figure 2 *A* shows the infection protection matrices for a combination of N and RBD antibody levels measured with Roche assays. The previously infected groups with the highest quartile of the Roche N index had extremely high protection irrespective of RBD antibody levels, ranging from 92% to 100%. In the previously infected groups with the lowest quartile of the Roche N index, higher RBD antibody levels were associated with greater protection: the protection (95% CI) from lowest to highest RBD quartile was −35% (−150% to 27%), 53% (11%–75%), 71% (39%–86%), and 87% (58%–96%), respectively (*P* < .01 for trend). In the infection-naive groups, higher anti-RBD titers correlated only slightly and not significantly with protection: the protection (95% CI) from the lowest to highest RBD quartile was 0 (reference), 34% (6%–54%), 37% (−1% to 60%), and 37% (−17% to 65%), respectively (*P* = .15 for trend). According to the contour plot of the continuous anti-RBD and N antibodies, high levels in either anti-RBD or N antibodies conferred relative protection, and high levels in both anti-RBD and N conferred further robust protection (Figure 2*B*). These results were similar in Abbott and Sysmex assays (Supplementary Figure 4).

## DISCUSSION

During the predominant wave of Omicron XBB.1.16/EG.5 in Japan, higher levels of pre-reinfection N antibodies were correlated with higher protection against reinfection irrespective of anti-RBD/S antibody levels among the individuals previously infected. Those with high levels of N and RBD/S antibodies had robust protection against reinfection. Among those with low levels of infection-acquired N antibodies, higher levels of vaccine-induced RBD/S antibodies conferred greater protection against reinfection.

The present finding showing higher protection against reinfection associated with higher pre-reinfection N antibodies among the individuals previously infected agrees with 2 previous studies conducted in Delta and Omicron BA.4/BA.5–predominant phases [8, 9] but not with the null association reported from 2 small studies during an earlier pandemic phase [3, 10]. Our study has some strengths over previous studies, including a larger sample size and a well-defined cohort. More important, we adjusted for baseline anti-RBD/S antibodies, enabling us to assess the independent role of N antibody levels. With these features, our study provides robust evidence of the association between N antibody levels and subsequent risk of reinfection with highly immune evasive Omicron BBX.1.16/EG.5 variants.

The present finding is supported by animal experiments suggesting an independent role of N antibodies against infection. In mouse models, anti-SARS-CoV-2 N antibodies have been shown to improve protection against SARS-CoV-2 challenge by eliciting natural killer–mediated antibody-dependent cellular cytotoxicity against infected cells [5, 19], which is another critical antibody response for protection besides neutralization [20]. In mice immunized with an N-specific vaccine, N antibody titer correlated with protection against SARS-CoV-2 challenge [6, 11]. Alternatively, N antibody titers might be just a surrogate marker for other components of infection-acquired immunity. For example, mucosal antibodies—which are largely induced by natural infection and play a critical role in protection against reinfection by preventing entry and spread of the virus in the upper respiratory tract [21, 22]—were reported to be modestly correlated with N antibodies among individuals with previous SARS-CoV-2 infection [23]. In addition, N-specific T cells have a pivotal role in protection against SARS-CoV-2 infection by the robust local and systemic interferon γ immune response [24, 25] and were shown to be modestly correlated with N antibodies among those with previous infection [26].

We first assessed the combined role of N and S/RBD antibody levels in relation to the risk of SARS-CoV-2 reinfection, showing an extremely high protection rate (>90%) among those high in N and S/RBD antibodies. Animal experiments demonstrated that vaccines encoding N and S proteins induced higher levels of these antibodies and conferred better protection against SARS-CoV-2 infection than vaccines encoding N or S protein alone [11, 12], suggesting that enhancing N-specific immunity coupled with S-specific immunity could enhance the protection. In humans, hybrid immunity (ie, vaccination and infection) shows a greater reduction in the risk of SARS-CoV-2 infection than vaccine-induced immunity alone [2], which could be partially explained by the joint effect of N and S/RBD antibodies.

In individuals who were previously infected, low N antibody titers represent long intervals since the infection [27], asymptomatic or mild symptoms at infection [28], or vaccination before the infection [27, 29]. In the present analysis, we found that a higher number of vaccine doses or shorter intervals from the last vaccination were associated with higher anti-S/RBD antibody titers and conferred greater protection against reinfection among individuals with low N antibodies who were previously infected. This result suggests that the additional vaccines enhance the protection against reinfection, even for those with a history of infection.

During the Omicron XBB.1.16/EG.5.1–dominant phase, the majority of SARS-CoV-2 infections (74%) occurred in participants who were infection naive at baseline, and their protection rate showed only 37% even in the highest quartile of Roche RBD titers, indicating a low protective ability of anti-RBD antibodies. Our result disagrees with previous studies conducted in pre-Omicron phases, which showed a robust correlation between the vaccine-induced anti-RBD antibodies and infection protection [30–32]. Yet, this discordance is reasonable, given that the anti-RBD antibodies of our infection-naive participants had been induced by the original monovalent vaccine or Omicron bivalent vaccine (original + Omicron BA.1 or BA.4/5), each of which has limited capacity to produce neutralizing antibodies against Omicron XXB.1.16 and EG.5.1 [33]. The Omicron XBB.1.5 monovalent vaccination, which started in autumn 2023 in several countries, including Japan, is expected to largely reduce the risk of SARS-CoV-2 infection through boosting the neutralization capacity against Omicron XXB variants more than the historical vaccines [34–36].

Strengths of this study include the following: a relatively large cohort size, a high participation rate (80%), the use of pre-infection antibody titers close to the timing of infection (ie, short follow-up), measures of N and S/RBD antibodies with 3 types of commercially available antibody assays, comprehensive adjustment for infection risk factors, and rigorous definition of previous and new infection with information on history of COVID-19 and at most 8 serologic test results since 2020. However, limitations should be acknowledged. First, we did not conduct active surveillance of SARS-CoV-2 infection during follow-up, possibly underestimating the number of total infections (eg, asymptomatic cases) [37]. In the sensitivity analysis restricting symptomatic cases, we confirmed that the association between N antibody levels and reinfection risk was virtually unchanged (Supplementary Table 3). Second, ascertaining SARS-CoV-2 infection by the in-house registry might not be completely recorded for nonregular staff. Nonetheless, we found a similar association between N antibody levels and reinfection risk after excluding nonregular staff (Supplementary Table 4). Third, since no severe cases were observed in the present study, the results do not apply to severe COVID-19. Fourth, we did not measure mucosal and cellular immunity, which was suggested to contribute to protection against SARS-CoV-2 infection [21, 22, 24, 25]. Fifth, we only partially measured the neutralizing antibodies, and these data were not included in the present analysis. Sixth, we cannot rule out the possibility that self-reported variables, such as infection prevention practices and infection risk behaviors, were influenced by social desirability and recall biases. Finally, given the lack of active surveillance and the short follow-up period, caution should be exercised to interpret the degree of protection that we estimated.

In conclusion, pre-reinfection N-specific antibody levels correlated with protection against reinfection after controlling for S-specific antibody levels among health care workers during the dominant wave of Omicron XBB.1.16 and EG.5 subvariants in Japan. Higher levels of N and S antibodies conferred robust protection. In individuals with low levels of N antibodies who were previously infected, vaccine-induced higher S antibody titer enhanced protection against reinfection. The level of N antibodies could have an independent role in infection protection and be a marker for deciding the timing of additional vaccination. Further research is warranted on whether vaccines encoding S and N proteins confer better protection against infection in humans.

## Supplementary Data

Supplementary materials are available at *Open Forum Infectious Diseases* online. Consisting of data provided by the authors to benefit the reader, the posted materials are not copyedited and are the sole responsibility of the authors, so questions or comments should be addressed to the corresponding author.

## Supplementary Material

ofae455_Supplementary_Data

## Notes

***Acknowledgments.*** We kindly appreciate Mika Shichishima for her contribution to data collection and the staff of the Laboratory Testing Department for their contribution to antibody testing.

***Author contributions.*** S. Y. and T. M. had full access to all data in the study and took responsibility for the integrity of the data and the accuracy of the data analysis. Conceptualization: S. Y., T. M., H. S., W. S., and N. O. Methodology: S. Y. and T. M. Software: S. Y. and M. K. Validation: M. K. and T. M. Formal analysis: S. Y. Investigation: S. Y., Y. O., N. I., T. N., T. T., K. H., K. O., M. K., and T. M. Resources: Y. O., T. M., H. S., N. A., W. S., and N. O. Data curation: S. Y., M. K., and T. M. Writing–original draft: S. Y. and T. M. Visualization: S. Y. Supervision: T. M. and N. O. Project administration: S. Y. and T. M. Funding acquisition: T. M. All authors contributed to manuscript revisions and editing.

***Patient consent statement.*** Written informed consent was obtained from all the participants. This study was approved by the NCGM Ethics Committee (NCGM-G-003598).

***Role of the funder/sponsor.*** The funding entities had no role in the design or conduct of the study; collection, management, analysis, and interpretation of the data; preparation, review, or approval of the manuscript; or the decision to submit the manuscript for publication.

***Financial support*.** This work was supported by the NCGM COVID-19 Gift Fund (grant 19K059); Japan Health Research Promotion Bureau Research Fund (grants 2020-B-09 and 2024-B-01); National Center for Global Health and Medicine (grants 21A2013D, 23A2020D, and 24A1011); and Abbott Japan and Roche Diagnostics, which provided reagents for the anti-SARS-CoV-2 antibody assays.
