# Supplementary material for: Correlates of Nucleocapsid Antibodies and a Combination of Spike and Nucleocapsid Antibodies Against Protection of SARS-CoV-2 Infection During the Omicron XBB.1.16/EG.5–Predominant Wave
Source: Open Forum Infect Dis. 2024 Aug 28;11(9):ofae455. doi: 10.1093/ofid/ofae455 (PMC11363870; doi:10.1093/ofid/ofae455)
Supplement: ofae455_Supplementary_Data [file ofae455_supplementary_data.zip › Supplemental Document 1.pdf]

2021 年 6 月作成（第 3 版）

この使用説明書をよく読んでから使用して下さい。

(研究用)HISCL SARS-CoV-2 N-IgG試薬

【全般的な注意】
1. 本品は研究用試薬です。この製品で得られた結果の臨床的有用性については確立されていないため、診断等の目的では使用しないでください。
2. 本使用説明書以外の使用方法については保証をいたしかねます。
3. 測定に使用する機器の添付文書および取扱説明書をよく読んでから使用してください。
4. 「2019-nCoV（新型コロナウイルス）感染を疑う患者の検体採取・輸送マニュアル」を参考にして、検体の取扱いや輸送を行ってください。
5. HISCL SARS-CoV-2 N-IgGキャリブレータの原料である血液は、60℃・1時間の加熱処理を行っていますが、感染の可能性を完全に否定できるものではありません。感染の危険性があるものとして、検体と同様に十分に注意して取扱いをしてください。

【キットの構成】
本キットは次の試薬により構成されています。

1	(研究用)HISCL SARS-CoV-2 N-IgG 試薬
	(研究用)HISCL SARS-CoV-2 N-IgG R1試薬
	(研究用)HISCL SARS-CoV-2 N-IgG R2試薬
	(研究用)HISCL SARS-CoV-2 N-IgG R3試薬
2	(研究用)HISCL SARS-CoV-2 N-IgG キャリブレータ
	(研究用)HISCL SARS-CoV-2 N-IgG C0
	(研究用)HISCL SARS-CoV-2 N-IgG C1
	(研究用)HISCL SARS-CoV-2 N-IgG C2
	(研究用)HISCL SARS-CoV-2 N-IgG C3
	(研究用)HISCL SARS-CoV-2 N-IgG C4

※上記の他、HISCL発光基質セット（HISCL R4試薬、HISCL R5試薬）及びHISCL洗浄液を使用します。

【使用目的】
血清又は血漿中のSARS-CoV-2核蛋白質に対するIgG型抗体の検出

【測定原理】
本法は、2ステップサンドイッチ法を用いた化学発光酵素免疫測定方法です。

【操作上の注意】
測定試料の性質、採取法
1. 検体は採取後、できるだけ速やかに測定してください。
2. 冷蔵又は冷凍保存されていた検体を使用する場合は室温に戻してください。

妨害物質・妨害薬剤
1. 濁りのある検体、溶血が見られる検体は、正しく測定が行えない恐れがあります。
2. ヘモグロビン(490mg/dL以下)、ビリルビン(ビリルビンF: 19.9mg/dL以下、ビリルビンC: 21.1mg/dL以下)、及び乳び(1,630ホルマジン濁度数以下)及びRF(440IU/mL以下)は記載の濃度以下では判定に影響を与えません。

その他
1. 本品は、「全自動免疫測定装置HISCL-5000およびHISCL-800」（シスメックス株式会社）の専用試薬であり、他の装置には使用できません。
2. 必ず本使用説明書で指定された試薬（キット構成参照）を使用してください。
3. R1～R3試薬は後述の測定（操作）法に従って融解してから使用してください。組立が不完全な場合、装置のエラーや試薬の蒸発が起こり、正しく測定が行えない恐れがあります。
4. R4試薬、R5試薬を装置にセットする際には体液中に広く含まれるアルカリホスファターゼの混入を防ぐため、手指の接触や唾液の飛散等に注意して取り扱ってください。またR5試薬はアルカリ性であり、空気中の二酸化炭素によるpH変動を避けるため、装置にセット後は交換時まで取り外さないでください。
5. 試料をサンプルカップ等に分注する場合は、蒸発の影響を考慮して200μL以上分注してください。なお、最低分注量については装置の取扱説明書をご覧ください。
6. キャリブレータは37℃で速やかに融解し、気泡が生じないようにゆるやかに混和してください。また、測定の際は6穴の検体ラックにアダプターを付けて、キャリブレータをセットしてください。

【用法・用量（操作方法）】
必要な器具・器材・試料等
・HISCL-5000又はHISCL-800
・消耗品
・反応キュベット、チップ等

測定（操作）法
1. 準備
(1) -20℃以下で保存された試薬は、使用前日から冷蔵下で静置し融解してください。
(2) R2試薬容器を取り出し、気泡が生じないようゆるやかに手振り攪拌し、磁性粒子が分散されたことを目視で確認してください。（転倒混和は避けてください。）

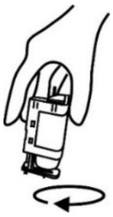

(3) R1/R3 試薬容器は、気泡が生じないようゆるやかに 5～10 回程度の転倒混和を行い、(2)の手振り攪拌後に 5 分程度静置してから使用ください。必ず、アルミシールを破る前に実施ください。

(4) 初回のみ、試薬容器前部の爪を押しながら容器ケースを完全に押し下げてください（アルミシールが破れて開栓されます）。

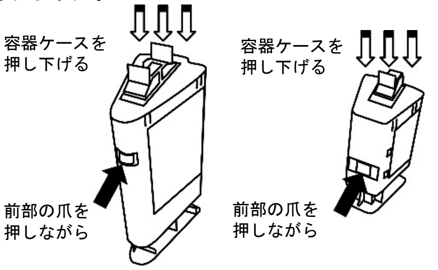

(5) 使用する装置の取扱説明書に従い、試薬容器を装置にセットしてください。

2. 標準操作法
(1) 反応キュベットにR1試薬120μLと試料10μLを分注し、42℃で2分間反応させます。
(2) R2試薬30μLを分注し、42℃で1分間反応させた後、磁気分離（反応キュベットに磁石を近づけ、液体部分を吸引除去）します。
(3) 洗浄液100～700μLの分注と磁気分離を組み合わせ洗浄します。この操作を計4回行います。
(4) R3試薬100μLを分注し、42℃で2.5分間反応させた後、磁気分離します。
(5) 洗浄液100～700μLの分注と磁気分離を組み合わせ洗浄します。この操作を計4回行います。
(6) R4試薬50μLを分注して混合攪拌した後、R5試薬100μLを分注して混合攪拌し、42℃で5分間反応させ、発光強度を測定します。

3. 検量線の作成
(1) HISCL SARS-CoV-2 N-IgG キャリブレータをフリーザから取り出し、37℃で速やかに融解し、気泡が生じないようにゆるやかに混和してください。
(2) 卓上遠心機でスピンダウン後、氷上に置いてください。
(3) キャリブレータは検体ラック上に右からC0～C4となるようにセットしてください。なお、キャリブレータの測定は、6穴の検体ラックにアダプターを付けたものをご使用ください。
(4) 装置の検量線画面から、検量線測定を選択し、ラック番号に6穴の検体ラック番号を設定してください。
(5) 項目は測定を実施する項目を選択し、方法はフルキャリブレーションを選択してください。
(6) R1/R2/R3、R4/R5、キャリブレータのロット番号が正確に入力されていることを確認してください。OKで次に進み、測定を開始してください。（なお、各濃度2回ずつ測定されます。）
(7) 各キャリブレータの発光強度を縦軸に、濃度を横軸にとり検量線を作成します。※

4. 検体測定
(1) 使用する装置の取扱説明書に従って検体をセットします。
(2) 2. 標準操作法に準じて測定を行い、発光強度を測定します。
(3) 発光強度を検量線に当てはめ、検体中のIgG型SARS-CoV-2抗体濃度を求めます。※
※装置ではこれらの操作を自動で行います。

【性能】
参考として、当該試薬ロットにて測定した結果を以下に示した。

1. 感度
(1) キャリブレータ(C0)を試料として測定した際、10,000カウント以下であった。

C0 発光強度
1,085
1,095
1,143

(2) キャリブレータ(10SU/mL付近)を試料として測定した際、10SU/mLの発光強度が800,000～3,100,000カウントの範囲であった。

10SU/mL あたりの発光強度
1,808,113
1,865,581
1,856,441

2. 正確性
管理用試料(L)および(H)を試料として測定した際、その定量値が表示値±20%以内であった。

Sample	管理用試料(L)	管理用試料(H)
表示値(SU/mL)	3.3	27.5
測定値(SU/mL)	3.3<br>3.2<br>3.2	26.3<br>27.6<br>27.7
mean(SU/mL)	3.2	27.2
SD(SU/mL)	0.1	0.8
CV	1.8%	2.9%
対 表示値	98.0%	98.9%
最大値(SU/mL)	3.3	27.7
対 表示値	100.0%	100.7%
最小値(SU/mL)	3.2	26.3
対 表示値	97.0%	95.6%

3. 同時再現性
管理用試料(L)および(H)を試料として10回 測定した際、そのCVは15%以内であった。

Sample	管理用試料(L)	管理用試料(H)
測定値(SU/mL)	3.3	26.3
	3.2	27.6
	3.2	27.7
	3.2	27.6
	3.3	27.8
	3.2	27.4
	3.2	27.4
	3.4	27.6
	3.3	27.0
	3.3	28.0
mean(SU/mL)	3.3	27.4
SD(SU/mL)	0.1	0.5
CV	2.1%	1.8%

【使用上又は取扱い上の注意】

取扱い上（危険防止）の注意

1. R1～R4試薬、HISCL SARS-CoV-2 N-IgGキャリブレータおよびコントロールには、アジ化ナトリウムが含有されていますが、法的には毒物として取り扱われません。また、R5試薬はアルカリ性（pH9.6）です。
これらの試薬が誤って目や口に入ったり皮膚に付着した場合は、水で十分に洗い流す等の応急処置を行い、必要があれば医師の手当て等を受けてください。
2. 検体はSARSコロナウイルス2やその他の感染性物質による感染の恐れがあるものとして、取扱いには厳重な注意をしてください。
3. 検査にあたっては感染の危険を避けるため使い捨て手袋等を着用してください。
4. 感染を避けるために口によるピペッティングを行わないでください。

使用上の注意

1. 各試薬は、気泡が生じないように、ていねいに扱ってください。気泡が生じると、測定が正常に行われないことがあります。この場合には、気泡が消えるのを待ってからご使用ください。
2. Lot No.が異なるR1～R3試薬を組み合わせて使用しないでください。また、Lot No.が同じであっても試薬をつぎ足して使用しないでください。使用期限を過ぎた試薬は使用しないでください。
3. R1～R3試薬を装置から取り出した場合は2～8℃で保存してください。装置に戻す場合はR2試薬容器を「用法・用量（操作方法）」に従って攪拌してからセットしてください。誤って凍結させた試薬は品質が変化して正しい結果が得られないことがありますので使用しないでください。
4. キャリブレータ及びコントロールは使い切りです。繰り返し使用することはできません。

廃棄上の注意

1. アジ化ナトリウムは、鉛、銅などと反応して爆発性の化合物を生成する危険性がありますので、廃棄の際には、大量の水と共に流してください。
2. 廃棄にあたっては水質汚濁防止法等の規制及び各都道府県の条例等に留意して処理してください。
3. 使用後の容器は、焼却処理するか、廃棄する場合には廃棄物に関する規定に従って医療廃棄物又は産業廃棄物等区別して処理してください。
4. 検体に接触した器具を滅菌する場合は、次のいずれかの方法で処理してください。
・0.05%ホルマリン溶液に37℃、72時間以上浸す。
・2%グルタルアルデヒド溶液に1時間以上浸す。
・次亜塩素酸ナトリウムを0.1%以上含む溶液に1時間以上浸す。
・121℃で少なくとも1時間以上オートクレーブにかける。
5. 検体、廃液等が飛散した場合は、2%グルタルアルデヒド溶液、次亜塩素酸ナトリウムを0.1%以上含む溶液等によるふき取りと消毒を行ってください。

その他の注意

1. 定期的な精度管理を実施してください。
2. 試薬の容器等は他の目的に転用しないでください。

【貯蔵方法・有効期間】

製品名	貯蔵方法	有効期間
(研究用)HISCL SARS-CoV-2 N-IgG試薬	（未開封）<br>-40℃以下で保存<br>-20℃以下で保存	 有効期限内<br>6カ月
	（開封後）<br>2～8℃で保存	10日間
(研究用)HISCL SARS-CoV-2 N-IgG<br>キャリブレータ	（未開封）<br>-40℃以下で保存<br>-20℃以下で保存	 有効期限内<br>6カ月
	（開封後）<br>2～8℃で保存	当日中
(研究用)HISCL SARS-CoV-2 N-IgG<br>コントロール	（未開封）<br>-40℃以下で保存<br>-20℃以下で保存	 有効期限内<br>6カ月
	（開封後）<br>2～8℃で保存	当日中

（参考）
HISCL発光基質セット（R4試薬、R5試薬）：2～8℃保存
HISCL洗浄液：2～30℃保存

【問合せ先】

シスメックス株式会社
〒651-0073　神戸市中央区脇浜海岸通1丁目5番1号
TEL(078)265-0500(代)
