# Supplementary material for: Correlates of Nucleocapsid Antibodies and a Combination of Spike and Nucleocapsid Antibodies Against Protection of SARS-CoV-2 Infection During the Omicron XBB.1.16/EG.5–Predominant Wave
Source: Open Forum Infect Dis. 2024 Aug 28;11(9):ofae455. doi: 10.1093/ofid/ofae455 (PMC11363870; doi:10.1093/ofid/ofae455)
Supplement: ofae455_Supplementary_Data [file ofae455_supplementary_data.zip › Supplemental Document 2.pdf]

4. R1~R4試薬、HISCL検体希釈液には、アジ化ナトリウムが含有されていますが、法的には毒物として取り扱われません。また、R5試薬はアルカリ性（pH9.6）です。これらの試薬が誤って目や口に入ったり皮膚に付着した場合は、水で十分に洗い流す等の応急処置を行い、必要があれば医師の手当て等を受けてください。

使用上の注意

1. 各試薬は、気泡が生じないように、ていねいに扱ってください。気泡が生じると、測定が正常に行われないことがあります。この場合には、気泡が消えるのを待ってからご使用ください。
2. Lot No.が異なるR1〜R3試薬を組み合わせて使用しないでください。また、Lot No.が同じであっても試薬をつぎ足して使用しないでください。使用期限を過ぎた試薬は使用しないでください。開封後の有効期間は30日です。
3. R1〜R3試薬を装置から取り出した場合は2〜8 ℃で保存してください。装置に戻す場合はR2試薬容器を【用法・用量（操作方法）】に従ってかくはんしてからセットしてください。
4. 誤って凍結させた試薬は品質が変化して正しい結果が得られないことがありますので使用しないでください。
5. 各キャリブレータは必要量を分注した後、速やかにふたをして2~8 ℃で保存してください。放置したままですと蒸発等の影響で濃度変化が起こり、キャリブレーションが正常に行えなくなります。
6. 検量線の有効期間は作成から30日です。ただし期間内でも、以下の場合には作成し直してください。
   - 新しいLot No.のR1〜R3試薬を使用する場合
   - 精度管理で異常が生じた場合
   - 装置の取扱説明書に記載されている特定のメンテナンス･修理を実施した場合

廃棄上の注意

1. アジ化ナトリウムは、鉛、銅などと反応して爆発性の化合物を生成する危険性がありますので、廃棄の際には、大量の水と共に流してください。
2. 廃棄にあたっては水質汚濁防止法等の規制及び各都道府県の条例等に留意して処理してください。
3. 使用後の容器は、焼却処理するか、廃棄する場合には廃棄物に関する規定に従って医療廃棄物又は産業廃棄物等区別して処理してください。
4. 検体に接触した器具を滅菌する場合は、次のいずれかの方法で処理してください。
   - 0.05 %ホルマリン溶液に37 ℃、72時間以上浸す。
   - 2 %グルタルアルデヒド溶液に1時間以上浸す。
   - 次亜塩素酸ナトリウムを0.1 %以上含む溶液に1時間以上浸す。
   - 121 ℃で少なくとも1時間以上オートクレーブにかける。
5. 検体、廃液等が飛散した場合は、2 %グルタルアルデヒド溶液、又は次亜塩素酸ナトリウムを0.1 %以上含む溶液等によるふき取りと消毒を行ってください。

その他の注意

1. 定期的な精度管理を実施してください。
2. 試薬の容器等は他の目的に転用しないでください。

【貯蔵方法・有効期間】

1. 貯蔵方法
   HISCL 洗浄液：2〜30 ℃で保存
   上記以外の構成試薬：2〜8 ℃で保存
2. 有効期間
   6カ月（使用期限は、外箱に表示しています。）

【包装単位】

製品名	構成試薬名	包装
(研究用)HISCL SARS-CoV-2 S-IgG試薬 100テスト	(研究用)HISCL SARS-CoV-2 S-IgG R1試薬※4	12 mL × 1
	(研究用)HISCL SARS-CoV-2 S-IgG R2試薬	3 mL × 1
	(研究用)HISCL SARS-CoV-2 S-IgG R3試薬※4	10 mL × 1
(研究用)HISCL SARS-CoV-2 S-IgG キャリブレータ※5	(研究用)HISCL SARS-CoV-2 S-IgG C0〜C4	5濃度 1 mL × 1
HISCL 発光基質セット※5	HISCL R4試薬	40 mL × 1
	HISCL R5試薬	70 mL × 1
HISCL 洗浄液※5	HISCL 洗浄液	10 L × 1

関連製品

製品名	構成試薬名	包装
HISCL検体希釈液※5	HISCL検体希釈液	20 mL × 1
(研究用)HISCL SARS-CoV-2 S-IgG コントロール※5	(研究用)HISCL SARS-CoV-2 S-IgG コントロール Level1	3 mL × 2
	(研究用)HISCL SARS-CoV-2 S-IgG コントロール Level2	3 mL × 2
	(研究用)HISCL SARS-CoV-2 S-IgG コントロール Level3	3 mL × 2

※4：R1試薬とR3試薬は一体型の容器で提供されます。
※5：これらの製品は別売品となります。

【主要文献】

(1) 社内データ
(2) Li K, Wu M, Huan B et al.: Dynamic changes in anti-SARS-CoV-2 antibodies during SARS-CoV-2 infection and recovery from COVID-19.: Nat Commun., **11**, 6044 (2020)
(3) Sethuraman N, Jeremiah SS, Ryo A.: Interpreting Diagnostic Tests for SARS-CoV-2.: JAMA., **323**, 2249(2020)
(4) 村田竜也、他: 大動脈解離に対する大動脈人工血管置換術後のHCV抗体陽転化現象の検討ー外科用接着剤による抗ウシ血清アルブミン抗体産生に伴う偽陽性反応に関してー，医学検査, **68**, 281 (2019).
(5) Paul A Kristiansen et al.: WHO International Standard for anti-SARS-CoV-2 immunoglobulin.: The Lancet, **397**, 1347(2021)
(6) Clinical and Laboratory Standards Institute (CLSI). Evaluation of Detection Capability for Clinical Laboratory Measurement Procedures; Approved Guideline—Second Edition. CLSI Document EP17-A2. Wayne, PA: CLSI; 2012.
(7) Clinical and Laboratory Standards Institute (CLSI). Evaluation of Precision Performance of Quantitative Measurement Methods; Approved Guideline—Second Edition. CLSI Document EP05-A2. Wayne, PA: CLSI; 2004.

【問合せ先】

シスメックス株式会社 カスタマーサポートセンター
神戸市西区室谷1丁目3番地の2 〒651-2241
Tel 0120-413-034

REF カタログ番号
LOT ロット番号
添付の文書参照
テスト数
保存温度
天地無用
使用期限

製造販売元
シスメックス株式会社
神戸市中央区脇浜海岸通1丁目5番1号 〒651-0073
Tel 078-265-0500

sysmex

2021年 8月作成（第1版）

この使用説明書をよく読んでから使用してください。

(研究用)HISCL™ SARS-CoV-2 S-IgG 試薬

【全般的な注意】

1. 本品は研究用試薬です。この製品で得られた結果の臨床的有用性については確立されていないため、診断等の目的では使用しないでください。
2. 本使用説明書以外の使用方法については保証をいたしかねます。
3. 測定に使用する機器の添付文書及び取扱説明書をよく読んでから使用してください。
4. 本品は、医療従事者や適切な訓練を受けた人が使用してください。

【形状・構造等（キットの構成）】

本キットは、次の試薬により構成されています。

1. (研究用)HISCL SARS-CoV-2 S-IgG 試薬
   (1) (研究用)HISCL SARS-CoV-2 S-IgG R1試薬（以下、R1試薬）
   (2) (研究用)HISCL SARS-CoV-2 S-IgG R2試薬（以下、R2試薬）
   SARS-CoV-2スパイクタンパク質固定化磁性粒子
   (3) (研究用)HISCL SARS-CoV-2 S-IgG R3試薬（以下、R3試薬）
   ALP融合抗ヒトIgGモノクローナル抗体
2. HISCL発光基質セット
   (1) HISCL R4試薬（以下、R4試薬）
   (2) HISCL R5試薬（以下、R5試薬）
   CDP-*Star*™
3. HISCL洗浄液（以下、洗浄液）
4. (研究用)HISCL SARS-CoV-2 S-IgG キャリブレータ（以下、キャリブレータ）
   (1) (研究用)HISCL SARS-CoV-2 S-IgG C0
   (2) (研究用)HISCL SARS-CoV-2 S-IgG C1
   (3) (研究用)HISCL SARS-CoV-2 S-IgG C2
   (4) (研究用)HISCL SARS-CoV-2 S-IgG C3
   (5) (研究用)HISCL SARS-CoV-2 S-IgG C4

ALP：アルカリホスファターゼ
CDP- *Star*™：
Disodium 2-chloro-5-(4-methoxyspiro{1,2-dioxetane-3,2'- (5'-chloro)-tricyclo[3.3.1.1 3,7]decan}-4-yl)-1-phenyl phosphate

【使用目的】

血清又は血漿中の新型コロナウイルス（SARS-CoV-2）のスパイクタンパク質を認識するIgG抗体（以下、SARS-CoV-2 S-IgG）の測定

【測定原理】

本法は、2ステップサンドイッチ法を用いた化学発光酵素免疫測定法です。

1. R1試薬と試料を混合します。
2. 試料中のSARS-CoV-2 S-IgGとR2試薬中のSARS-CoV-2スパイクタンパク質固定化磁性粒子が特異的に反応します。
3. 未反応液を除去後、R3試薬を添加すると、ALP融合抗ヒトIgGモノクローナル抗体が磁性粒子上のSARS-CoV-2 S-IgGと特異的に反応します。
4. 未反応液を除去後、R4試薬及びR5試薬を添加すると、発光基質CDP-*Star*™が磁性粒子上のALPにより分解され、生じた発光の強度を測定します。試料中のSARS-CoV-2 S-IgG濃度に応じて発光強度が増加しますので、あらかじめ既知濃度のSARS-CoV-2 S-IgGを含む試料（(研究用)HISCL SARS-CoV-2 S-IgG キャリブレータ）を測定して検量線を作成しておくことにより、試料中のSARS-CoV-2 S-IgG濃度を求めることができます。

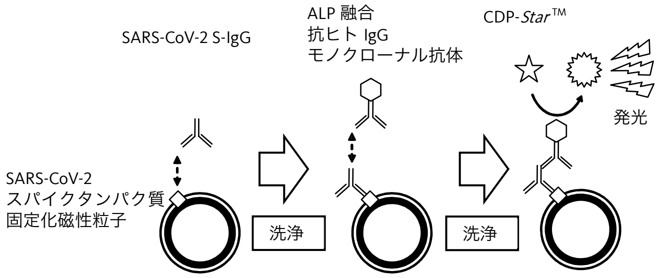

【操作上の注意】

測定試料の性質，採取法

1. ヒトの血清、血漿以外について本キットの性能は保証していません。
2. 検体の採取は使用する採血管の添付文書をよく確認し、指定された方法（採血量、遠心分離など）により採取してください。
3. 検体は採取後、できるだけ速やかに測定してください。可能な限り新鮮な検体を用いてください。
4. 検体の保存が必要な場合は、2~8 ℃で3日間、30 ℃で8時間、それ以上の場合は-20 ℃以下で凍結して保存してください。
   なお、2~8 ℃で3日間又は、30 ℃で8時間保存したところ、値の変動はいずれも10 %以内でした。
5. 保存された検体は測定前によく混合等してください。なお、凍結保存した検体は混合前に、完全に融解していることを確認してください。
6. 検体を保存する際は、サンプルカップは使用せず、必ず密閉できる容器を用いてください。
7. 「2019-nCoV（新型コロナウイルス）感染を疑う患者の検体採取・輸送マニュアル」を参考にして、検体の取扱いや輸送を行ってください。
8. 血清又は血漿検体を使用する場合は以下を使って採血されたものを使用してください。液体の抗凝固剤を使用すると、検体が希釈されて測定結果が低めになる可能性があります。

検体種	採血管
血清	血清
	血清分離剤入り
血漿	EDTA・2K
	EDTA・3K
	EDTA・2Na
	ヘパリン・Na
	ヘパリン・Li

9. 同一被験者で経過観察する際は、同じ種類の採血管を使用してください。
10. 検体固有の性質により、まれに希釈直線性が得られない場合があります。
11. 検体に気泡が発生している場合、検体吸引が正常に行われない場合があるため、必ず気泡を取り除いてから測定してください。
12. 加熱不活化処理した検体は、使用しないでください。

妨害物質・妨害薬剤

1. フィブリン塊等の固形物が見られる検体は、2,000 ×gで10分間以上遠心分離し、固形物を除去してから測定してください。
2. 濁りのある検体、溶血が見られる検体は、正しく測定が行えない恐れがあります。

3. 下表に示す物質が本品の測定に与える影響を確認したところ、下表の各濃度までは測定に与える値の変動は10 %以下でした。

物質	濃度
ヘモグロビン	494.8 mg/dL
ビリルビンF	19.3 mg/dL
ビリルビンC	20.9 mg/dL
乳ビ	1,610.3 FTU
RF	550.8 IU/mL
ビオチン（ビタミンB_7）	3,499.1 ng/mL

※ ここに記載したデータは代表的な例であり、各施設では異なる結果を示す場合があります。

その他

1. 本品は「全自動免疫測定装置HISCL-5000」(シスメックス株式会社)又は同等の性能を有する専用機器の専用試薬であり、他の装置には使用できません。
2. 必ず使用説明書で指定された試薬（R1～R5試薬・キャリブレータ・洗浄液等）を使用してください。
3. R1～R3試薬容器は、後述の測定（操作）法に従って正しく組み立ててから使用してください。組立が不完全な場合、装置のエラーや試薬の蒸発が起こり、正しく測定が行えない恐れがあります。
4. R4試薬、R5試薬を装置にセットする際には、体液中に広く含まれるアルカリホスファターゼの混入を防ぐため、手指の接触や唾液の飛散等に注意して取り扱ってください。またR5試薬はアルカリ性であり、空気中の二酸化炭素によるpH変動を避けるため、セット後は交換時まで取り外さないでください。
5. 試料をサンプルカップ等に分注する場合は、蒸発の影響を考慮して速やかに測定してください。なお、最低分注量については、各装置の取扱説明書をご確認ください。

【用法・用量（操作方法）】

試薬の調製方法

本キットの各構成試薬及びHISCL検体希釈液（トリエタノールアミン緩衝液、BSA等）は調製済みですので、そのまま使用してください。

必要な器具・器材・試料等

・ マイクロピペット、サンプリングチップ及びサンプルカップ
・ HISCL-5000又は同等品
　本品はHISCL-2000*i*では使用できません。
・ HISCL反応キュベット又は同等品
・ HISCLチップ

測定（操作）法

1. 準備
 (1) R2試薬容器を取り出し、気泡が生じないようゆるやかに手振りかくはんし、磁性粒子が分散されたことを目視で確認してください（転倒混和は避けてください）。

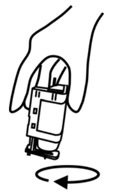

 (2) 初回のみ、試薬容器前部の爪を押しながら容器ケースを完全に押し下げてください（アルミシールが破れて開栓されます）。

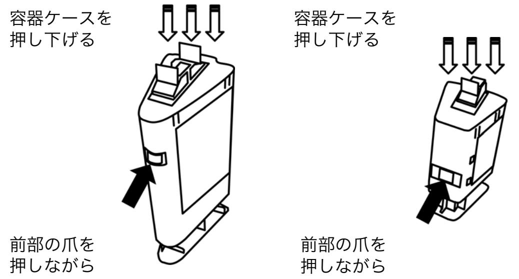

 (3) 使用する装置の取扱説明書に従い、試薬容器を装置にセットしてください。
2. 標準操作法※1
 (1) 反応キュベットにR1試薬120 μLと試料10 μLを分注し、42 ℃で3分間反応させます。
 (2) R2試薬を30 μL分注し、42 ℃で2分間反応させた後、磁気分離（反応キュベットに磁石を近づけ、液体部分を吸引除去）します。
 (3) 洗浄液200～900 μLの分注と磁気分離を組合せ洗浄します。この操作を計4回行います。
 (5) R3試薬100 μLを分注し、42 ℃で3分間反応させた後、磁気分離します。
 (6) 洗浄液200～900 μLの分注と磁気分離を組合せ洗浄します。この操作を計4回行います。
 (7) R4試薬50 μLを分注して混合かくはんした後、R5試薬100 μLを分注して混合かくはんし、42 ℃で5.5分間反応させ、発光強度を測定します。
3. 検量線の作成
 (1) (研究用)HISCL SARS-CoV-2 S-IgG キャリブレータ C0～C4（別売品）のそれぞれを、泡立たないように静かにかくはんし、使用する装置の取扱説明書に従ってセットします。
 (2) 2.標準操作法に準じて測定を行い、発光強度を測定します。
 (3) 各キャリブレータの発光強度を縦軸に、濃度を横軸にとり、検量線を作成します。※1
4. 検体の測定
 (1) 使用する装置の取扱説明書に従って検体をセットします。
 (2) 2.標準操作法に準じて測定を行い、発光強度を測定します。必要に応じてHISCL検体希釈液を用いて装置内で40倍に自動希釈し、測定試料とすることもできます。
 (3) 発光強度を検量線に当てはめ、検体中のSARS-CoV-2 S-IgG濃度を求めます。※1

※1：装置ではこれらの操作を自動で行います。

5. 精度管理
 (1) (研究用)HISCL SARS-CoV-2 S-IgG コントロール Level1～3(別売品)をサンプルカップに分注します。最低分注量については、各装置の取扱説明書をご確認ください。
 (2) 2.標準操作法に準じて測定を行い、発光強度を測定します。
 (3) 発光強度を検量線に当てはめ、SARS-CoV-2 S-IgG抗体濃度を求めます。
 (4) (3)の測定値が許容範囲内にあることを確認します。
 ※精度管理は少なくとも24時間に1回の実施を推奨しています。コントロールの測定値が許容範囲内にあることを確認してから、検体測定を行ってください。

【測定結果の判定法】

判定法

カットオフ値：20.0 BAU/mL[(1)]

判定上の注意

1. SARS-CoV-2に対するIgG抗体は、一般的に初期感染数日後で血液中に検出されますが、感染後に存在する抗体の持続時間は十分なエビデンスが得られていません。
2. 抗体陽転後数週間にわたってウイルスが検出されることがあります[(2)(3)]。
3. 母集団によりカットオフ値が異なる場合がありますので施設ごとに設定してください。
4. 免疫反応を用いた測定を行う場合、非特異反応が起こり得ますので測定結果に基づく解釈は他の検査及び臨床症状等を考慮して総合的に判断してください。
 非特異反応の原因としては、各種の自己抗体、不溶物（特にフィブリン）及び自然抗体などが考えられます。
5. 原倍測定での測定結果が測定範囲上限を超え、更に高濃度域の測定結果を得る必要がある場合は、装置にて40倍希釈を実施して測定してください。また自動希釈(40倍)で測定範囲下限未満の場合は検体を希釈せず、原倍で測定してください。
6. ウシ血清アルブミン（bovine serum albumin; BSA）を含む薬剤を投与された患者では、血中に抗BSA抗体が産生されることがあるため、試薬中のBSAに結合し、正しい測定値が得られない可能性があります。測定結果に基づく解釈は他の検査及び臨床症状等を考慮して総合的に判断してください[(4)]。

【性能】

性能

ここに記載したデータは代表的な例であり、各施設では異なる結果を示す場合があります。

1. 感度
 (1) (研究用)HISCL SARS-CoV-2 S-IgG C0を試料として測定した場合の発光強度は、5,000 カウント※2以下です。
 (2) (研究用)HISCL SARS-CoV-2 S-IgG C0及び(研究用)HISCL SARS-CoV-2 S-IgG C1を試料として測定した場合の10 BAU/mLあたりの発光強度の比は、30 以上です。
 ※10 BAU/mLあたりの発光強度の比＝
 （(研究用)HISCL SARS-CoV-2 S-IgG C1の発光強度 －（研究用)HISCL SARS-CoV-2 S-IgG C0の発光強度）÷ (研究用)HISCL SARS-CoV-2 S-IgG C1表示濃度 × 10 ÷ (研究用)HISCL SARS-CoV-2 S-IgG C0の発光強度
2. 正確性
 SARS-CoV-2 S-IgG管理用試料※3を測定するとき、測定値はそれぞれ既知濃度の 100±20 %の範囲内です。
3. 同時再現性
 SARS-CoV-2 S-IgG管理用試料※3をそれぞれ10回同時に測定するとき、測定値のCV値は、10 %以下です。
4. 測定範囲
 5.0～500.0 BAU/mL（原倍測定時）
 200.0～20,000.0 BAU/mL（自動希釈(40倍)時の測定範囲）
 なお、自動希釈（1,600倍）時の測定値は参考値とします。
 ※2：カウント：HISCL専用装置の発光強度の単位
 ※3：管理用試料：
 ここで用いるSARS-CoV-2 S-IgG管理用試料（低値、中値、高値）は、SARS-CoV-2 S-IgG陰性である溶液にSARS-CoV-2 S-IgG高濃度血漿を添加して調製したものです。
 SARS-CoV-2 S-IgG低値管理用試料　16.0～24.0 BAU/mL
 SARS-CoV-2 S-IgG中値管理用試料　160.0～240.0 BAU/mL
 SARS-CoV-2 S-IgG高値管理用試料　320.0～480.0 BAU/mL

相関性

血清検体と血漿検体との相関性を検討したところ、77例の検体において良好な相関性が得られました。

 相関係数 r=0.995
 回帰式　y=0.985X + 0.112
 y：血漿検体、x：血清検体

較正用基準物質に関する情報

First WHO International Standard for anti-SARS-CoV-2 immunoglobulin (human) NIBSC code:20/136 [(5)]

1. 検出限界・定量限界
 CLSIガイドラインEP17-A2[(6)]に従って、ブランク限界（LoB）、検出限界（LoD）及び定量限界（LoQ）をHISCL-5000にて算出しました。

	BAU/mL
LoB	0.17
LoD	0.19
LoQ	0.51

2. 精度
 CLSIガイドラインEP05-A2[(7)]に従って、併行精度（CV_r）、室内再現精度（CV_T）、室間再現精度（CV）をHISCL-5000にて求めました。

標本	N	平均値 (BAU/mL)	併行精度	
			SD	CV_r
SARS-CoV-2 S-IgG 低値管理用試料	80	21.4	0.62	2.9 %
SARS-CoV-2 S-IgG 中値管理用試料	80	204.5	4.63	2.3 %
SARS-CoV-2 S-IgG 高値管理用試料	80	392.2	7.10	1.8 %

標本	N	平均値 (BAU/mL)	室内再現精度	
			SD	CV_T
SARS-CoV-2 S-IgG 低値管理用試料	80	21.4	0.77	3.6 %
SARS-CoV-2 S-IgG 中値管理用試料	80	204.5	8.17	4.0 %
SARS-CoV-2 S-IgG 高値管理用試料	80	392.2	12.71	3.2 %

標本	n	平均値 (BAU/mL)	室間再現精度	
			SD	CV
SARS-CoV-2 S-IgG 低値管理用試料	75	20.5	0.80	3.9 %
SARS-CoV-2 S-IgG 中値管理用試料	75	193.6	8.01	4.1 %
SARS-CoV-2 S-IgG 高値管理用試料	75	373.5	16.38	4.4 %

3. 特異性
 3.1 健常者検体
 2019年12月以前の健常者検体477例をHISCL-5000にて測定しました。

	n	SARS-CoV-2 S-IgG		特異性
		陰性判定	陽性判定	
健常者検体	477	476	1	99.8 %

 3.2 その他の疾患等

	n	SARS-CoV-2 S-IgG	
		陰性判定	陽性判定
インフルエンザA	5	5	0
インフルエンザB	2	2	0
HBV	5	5	0
HCV	5	5	0
HIV	5	5	0
HTLV-1	5	5	0
全身性エリテマトーデス	2	2	0
グレーブス病	5	5	0
抗核抗体（ANA）	5	5	0
シェーグレン症候群	5	5	0
関節リウマチ	5	5	0
HAMA	5	5	0

 また、抗SARS-CoV-2モノクローナル抗体溶液（S-IgM、N-IgGおよびN-IgM）をHISCL-5000にて測定したところ、測定結果はカットオフ値以下でした。

モノクローナル抗体カテゴリー	添加濃度（μg/mL）
SARS-CoV-2 S-IgM	9.12
SARS-CoV-2 N-IgG	9.37
SARS-CoV-2 N-IgM	9.08

4. 感度
 臨床診断でSARS-CoV-2陽性と確認された患者から採取された99例の血漿検体をHISCL-5000にて測定しました。

発症後日数	n	SARS-CoV-2 S-IgG		感度
		陰性判定	陽性判定	
≧21日	99	0	99	100 %

【使用上又は取扱い上の注意】

取扱い上（危険防止）の注意

1. 検体は、SARS-CoV-2, HBV, HCV, HIV等による感染の恐れがあるものとして、取扱いには厳重な注意をしてください。誤って目や口に入ったり、皮膚に付着した場合は、水で十分に洗い流す等の応急処置を行い、必要があれば医師の手当て等を受けてください。院内感染対策マニュアル等がある場合はその指示に従ってください。
2. 検査にあたっては感染の危険を避けるため使い捨て手袋、マスク等を着用してください。
3. 感染を避けるために口によるピペッティングを行わないでください。
