# Supplementary material for: Correlates of Nucleocapsid Antibodies and a Combination of Spike and Nucleocapsid Antibodies Against Protection of SARS-CoV-2 Infection During the Omicron XBB.1.16/EG.5–Predominant Wave
Source: Open Forum Infect Dis. 2024 Aug 28;11(9):ofae455. doi: 10.1093/ofid/ofae455 (PMC11363870; doi:10.1093/ofid/ofae455)
Supplement: ofae455_Supplementary_Data [file ofae455_supplementary_data.zip › Supplementary Materials.docx]

**Supplementary Materials**

Correlates of nucleocapsid antibodies and a combination of spike and nucleocapsid antibodies against protection of SARS-CoV-2 infection during Omicron XBB.1.16 and EG.5 predominant wave

Shohei Yamamoto, Yusuke Oshiro, Natsumi Inamura, Takashi Nemoto, Tomofumi Tan, Kumi Horii, Kaori Okudera, Maki Konishi, Tetsuya Mizoue, Haruhito Sugiyama, Nobuyoshi Aoyanagi, Wataru Sugiura, Norio Ohmagari

[**Supplemental Figure 1.** Venn diagram of diagnostic history and N-antibody status among participants defined as previous infection. N: nucleocapsid antibody 2](#_Toc169781979)

[**Supplemental Table 1.** Hazard ratios (95% confidence intervals) for subsequent SARS-CoV-2 infection across the baseline anti-nucleocapsid antibody index 3](#_Toc169781980)

[**Supplemental Figure 2.** Association between anti-SARS-CoV-2 nucleocapsid antibody level and risk of reinfection among previously infected individuals 4](#_Toc169781981)

[**Supplemental Figure 3.** Scatter plots between anti-nucleocapsid and anti-spike/RBD antibodies with each histogram among previously infected individuals 5](#_Toc169781982)

[**Supplemental Table 2.** Association between vaccination status and anti-RBD antibodies by the anti-nucleocapsid antibody status 6](#_Toc169781983)

[**Supplemental Figure 4.** Infection protection matrices for the baseline anti-nucleocapsid and anti-spike/RBD antibody levels 7](#_Toc169781984)

[**Supplemental Table 3.** Hazard ratios (95% confidence intervals) for subsequent “symptomatic” SARS-CoV-2 infection across the baseline anti-nucleocapsid antibody index 8](#_Toc169781985)

[**Supplemental Table 4.** Hazard ratios (95% confidence intervals) for subsequent SARS-CoV-2 infection across the baseline anti-nucleocapsid antibody index among participants, excluding non-regular staff (N=2,414) 9](#_Toc169781986)

**
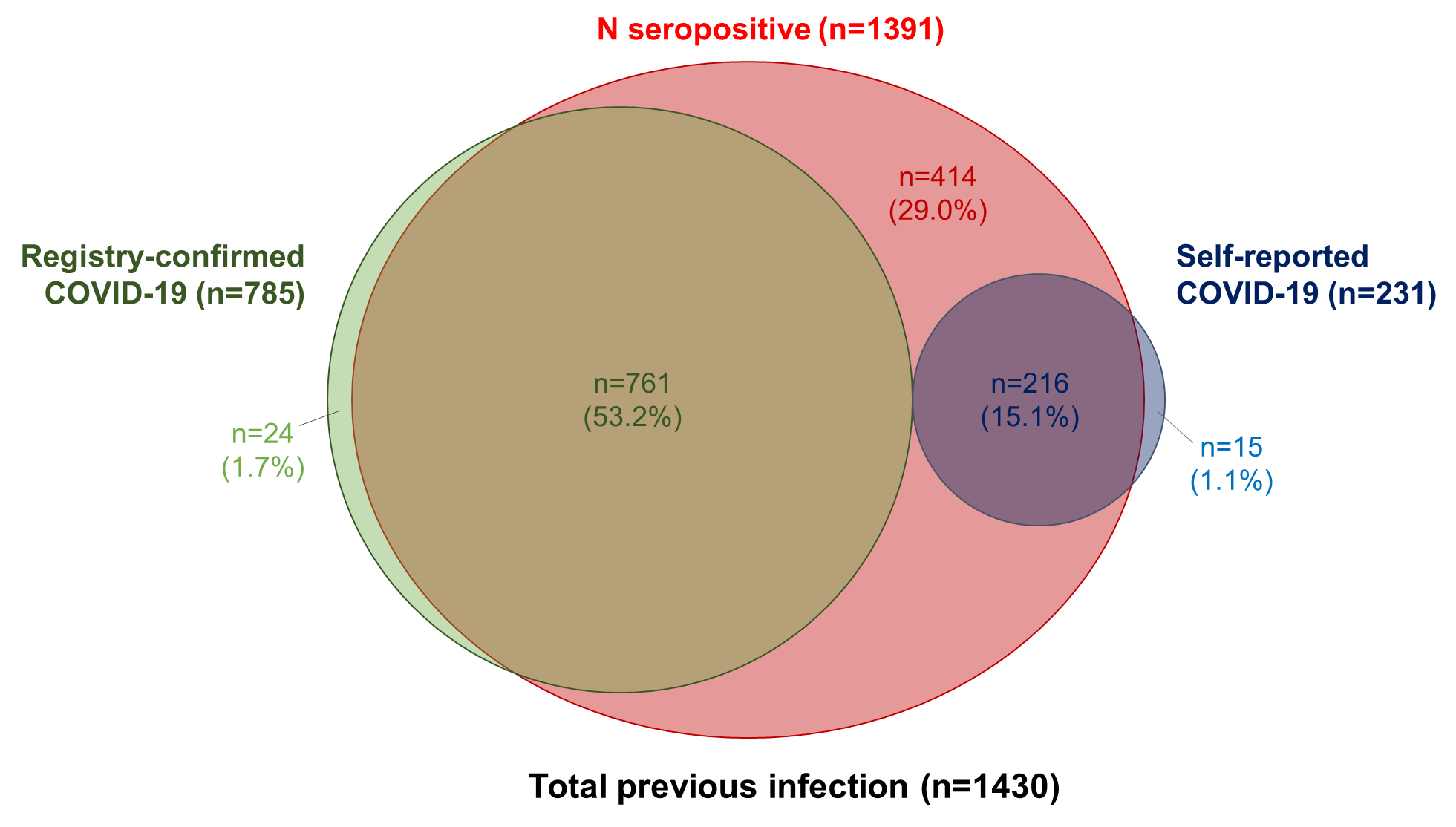
**

**Supplemental Figure 1.** Venn diagram of diagnostic history and N-antibody status among participants defined as previous infection. N: nucleocapsid antibody

**Supplemental Table 1.** Hazard ratios (95% confidence intervals) for subsequent SARS-CoV-2 infection across the baseline anti-nucleocapsid antibody index

| **Assays** | **Infection-naïve** | **Quartile of N antibodies among previously infected individuals** | | | | ***P* for trend** among previously infected individuals |
| --- | --- | --- | --- | --- | --- | --- |
|  |  | **Q1 (lowest)** | **Q2** | **Q3** | **Q4 (highest)** |  |
| **Abbott (IgG N)** |  |  |  |  |  |  |
| S/C, median [min–max] | 0.06 [0.01–1.29] | 0.12 [0.01–0.22] | 0.36 [0.23–0.56] | 0.84 [0.57–1.31] | 2.55 [1.32–10.7] |  |
| Cases/Person-days (% ^a^) | 175/71901 (24.3) | 26/26150 (9.9) | 16/25121 (6.4) | 13/26620 (4.9) | 7/26205 (2.7) |  |
| Model 1 | 2.69 (1.78–4.06) | reference | 0.64 (0.34–1.19) | 0.48 (0.25–0.93) | 0.27 (0.12–0.61) | <0.01 |
| Model 2 | 2.78 (1.82–4.24) | reference | 0.66 (0.35–1.23) | 0.49 (0.25–0.95) | 0.27 (0.12–0.63) | <0.01 |
| Model 3 | 2.36 (1.54–3.64) | reference | 0.71 (0.38–1.33) | 0.55 (0.28–1.08) | 0.37 (0.16–0.87) | 0.04 |
| **Sysmex (IgG N)** |  |  |  |  |  |  |
| SU/mL, median [min–max] | 0.1 [0–8.4] | 2.6 [0–5.1] | 8.5 [5.2–13.6] | 21.7 [13.7–34.2] | 97.4 [34.8–94024] |  |
| Cases/Person-days (% ^a^) | 175/71901 (24.3) | 33/25107 (13.1) | 18/25944 (6.9) | 7/26395 (2.7) | 4/26650 (1.5) |  |
| Model 1 | 2.04 (1.40–2.97) | reference | 0.52 (0.29–0.93) | 0.20 (0.09–0.45) | 0.11 (0.04–0.32) | <0.01 |
| Model 2 | 2.06 (1.41–3.03) | reference | 0.51 (0.29–0.90) | 0.20 (0.09–0.45) | 0.12 (0.04–0.33) | <0.01 |
| Model 3 | 1.86 (1.26–2.75) | reference | 0.53 (0.30–0.94) | 0.21 (0.09–0.49) | 0.14 (0.05–0.40) | <0.01 |

Shown are the hazard ratios (95% confidence intervals).

Model 1 was adjusted for age (continuous) and sex (male or female).

Model 2 was additionally adjusted for job (doctors, nurses, allied health professionals, researchers, administrative staff, or others), occupational SARS-CoV-2 exposure risk (low, moderate, or high), body mass index (continuous), comorbid diseases (no or yes), immunosuppression (no or yes), use of tobacco products (no or yes), frequency of alcohol drinking (none, occasional, or weekly/daily drinker), number of household members (continuous), infection prevention score (continuous), spending ≥30 min in the 3Cs without mask (none, 1–5 times, or ≥6 times), and having dinner in a group of ≥5 people for >1 hours (none, 1–5 times, or ≥6 times).

Model 3 was further adjusted for anti-spike/RBD titers measured with the assay of the same company.

^a^ Incident rate per 10000 person-day

*Abbreviations*: 3Cs, crowded places, close-contact settings, and confined and enclosed spaces; IgG, immunoglobulin G; N, nucleocapsid; Q, quartile; S/CO, signal to cut-off; SU, Sysmex unit.


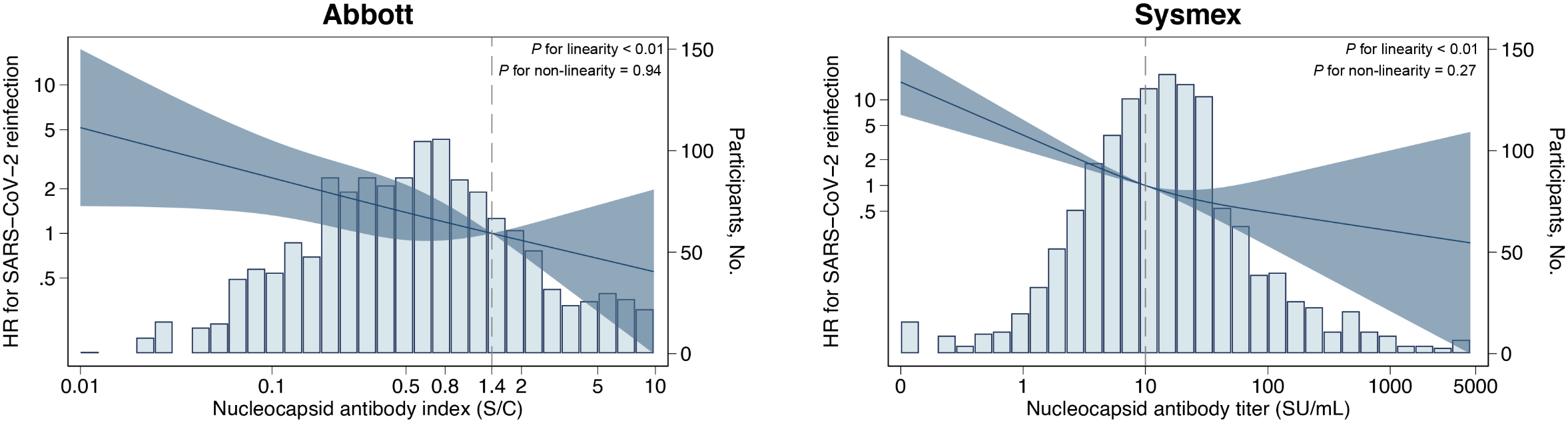


**Supplemental Figure 2.** Association between anti-SARS-CoV-2 nucleocapsid antibody level and risk of reinfection among previously infected individuals

Solid lines indicate the hazard ratio for SARS-CoV-2 reinfection, and the shaded area represents 95% confidence intervals. The bars indicate histograms of log-transformed nucleocapsid antibody levels. Reference points are seropositive thresholds for the each assay (1.4 S/C for Abbott, and 10.0 SU/mL for Sysmex). All models were adjusted for covariates of Model 2 in Table 2.

*Abbreviations*: IgG, immunoglobulin G; N, nucleocapsid; Q, quartile; S/CO, signal to cut-off; SU, Sysmex unit.

**Supplemental Figure 3.** Scatter plots between anti-nucleocapsid and anti-spike/RBD antibodies with each histogram among previously infected individuals

*Abbreviations*: AU, arbitrary units; COI, cut-off index; IgG, immunoglobulin G; N, nucleocapsid; Q, quartile; RBD, receptor-binding domain; S/CO, signal to cut-off; SU, Sysmex unit.

**Supplemental Table 2.** Association between vaccination status and anti-RBD antibodies by the anti-nucleocapsid antibody status

| **Vaccination status** | **Quartile of anti-RBD total titer (Roche, U/mL)** | | | | **P for trend** |
| --- | --- | --- | --- | --- | --- |
|  | **S-Q1** | **S-Q2** | **S-Q3** | **S-Q4** |  |
| **Infection naïve** | | | | | |
| ≤2-dose | 8.7 | 0.3 |  |  | <0.01 |
| 3-dose | 23.2 | 10.8 | 6.4 | 1.9 | <0.01 |
| 4-dose | 42.1 | 42.8 | 31.8 | 25.0 | <0.01 |
| 5-dose | 26.0 | 46.1 | 61.8 | 73.1 | <0.01 |
| Interval from last vaccination, days | 294 (189–455) | 200 (177–295) | 182 (172–216) | 175.5 (67–185) | <0.01 |
| **N-Q1: previously infected** | | | | | |
| ≤2-dose | 17.0 | 3.2 | 4.1 |  | <0.01 |
| 3-dose | 34.0 | 31.6 | 22.0 | 16.1 | <0.01 |
| 4-dose | 36.2 | 44.2 | 48.0 | 41.9 | 0.58 |
| ≥5-dose | 12.8 | 21.1 | 26.0 | 41.9 | <0.01 |
| Interval from last vaccination, days | 310 (242–523) | 294 (181–510) | 216 (182–304) | 209 (180–298) | <0.01 |
| **N-Q2: previously infected** | | | | | |
| ≤2-dose | 22.2 | 2.4 | 3.0 | 1.0 | <0.01 |
| 3-dose | 36.1 | 36.9 | 25.6 | 15.4 | <0.01 |
| 4-dose | 36.1 | 53.6 | 46.6 | 43.3 | 0.82 |
| ≥5-dose | 5.6 | 7.1 | 24.8 | 40.4 | <0.01 |
| Interval from last vaccination, days | 298 (269–516) | 298 (216–463) | 288 (180–455) | 191.5 (177–295) | <0.01 |
| **N-Q3: previously infected** | | | | | |
| ≤2-dose | 28.6 | 6.1 | 1.8 | 4.3 | <0.01 |
| 3-dose | 23.8 | 43.9 | 32.7 | 23.6 | 0.03 |
| 4-dose | 38.1 | 33.3 | 44.5 | 42.2 | 0.34 |
| ≥5-dose | 9.5 | 16.7 | 20.9 | 29.8 | <0.01 |
| Interval from last vaccination, days | 336 (294–514) | 306 (286–542) | 292.5 (186–462) | 250 (181–309) | <0.01 |
| **N-Q4: previously infected** | | | | | |
| ≤2-dose | 38.5 | 3.4 | 3.1 |  | <0.01 |
| 3-dose | 11.5 | 34.5 | 31.6 | 22.9 | 0.69 |
| 4-dose | 34.6 | 48.3 | 45.9 | 44.0 | 0.82 |
| ≥5-dose | 15.4 | 13.8 | 19.4 | 33.1 | <0.01 |
| Interval from last vaccination, days | 285 (181–511) | 296 (194–456) | 298 (220–462) | 284 (181–310) | <0.01 |

Data are presented as percentages for categorical variables and median (interquartile range) for continuous variables.

P for trend was calculated by the Cochran–Armitage test for the categorical variables and by the Jonckheere–Terpstra test for the continuous variables.

Q, quartile; N, nucleocapsid; S, spike


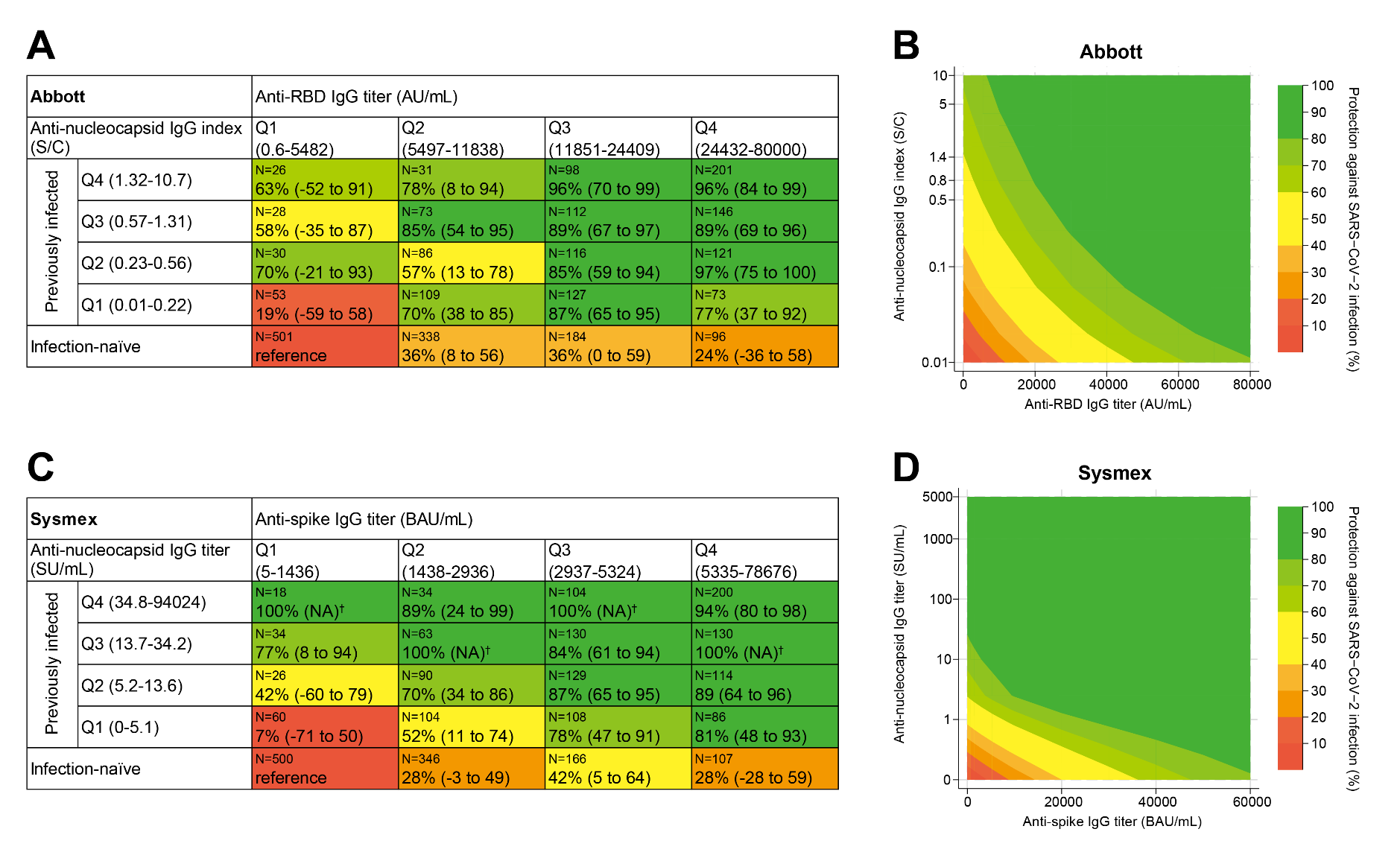


**Supplemental Figure 4.** Infection protection matrices for the baseline anti-nucleocapsid and anti-spike/RBD antibody levels

Matrix tables of the protection against infection by categorical anti-nucleocapsid and anti-spike/RBD antibodies, relative to the reference group of infection-naïve and lowest quartile of anti-spike/RBD antibodies, with Abbott (A) and Sysmex (C) assays. Also shown are contour plots of the protection by continuous anti-nucleocapsid and anti-spike/RBD antibodies, relative to reference values of the lowest value of anti-nucleocapsid and anti-spike/RBD antibodies, with Abbott (B) and Sysmex (D) assays.

Protection was calculated as (1 – hazard ratio) × 100. The hazard ratio was estimated using a Cox proportional hazards regression model, adjusting covariates of Model 2 in Table 2.

*: P<0.05

†: No incidence of subsequent SARS-CoV-2 infection in the group

Abbreviations: AU, arbitrary units; IgG, immunoglobulin G; N, nucleocapsid; Q, quartile; RBD, receptor-binding domain; S/CO, signal to cut-off; SU, Sysmex unit.

**Supplemental Table 3.** Hazard ratios (95% confidence intervals) for subsequent “symptomatic” SARS-CoV-2 infection across the baseline anti-nucleocapsid antibody index

| **Assays** | **Infection-naïve** | **Quartile of N antibodies among previously infected individuals** | | | | ***P* for trend** among previously infected individuals |
| --- | --- | --- | --- | --- | --- | --- |
|  |  | **Q1 (lowest)** | **Q2** | **Q3** | **Q4 (highest)** |  |
| **Roche (Total N)** |  |  |  |  |  |  |
| COI, median [min–max] | 0.08 [0.07–0.98] | 1.16 [0.07–2.46] | 4.30 [2.48–7.14] | 11.3 [7.15–20.7] | 48.9 [20.8–256] |  |
| Cases/Person-days (% ^a^) | 169/71901 (23.5) | 33/24958 (13.2) | 7/25897 (2.7) | 11/26564 (4.1) | 5/26677 (1.9) |  |
| Model 1 | 1.91 (1.31–2.77) | reference | 0.19 (0.08–0.43) | 0.30 (0.15–0.59) | 0.14 (0.05–0.35) | <0.01 |
| Model 2 | 1.93 (1.32–2.84) | reference | 0.19 (0.08–0.44) | 0.29 (0.15–0.58) | 0.14 (0.05–0.36) | <0.01 |
| Model 3 | 1.57 (1.05–2.33) | reference | 0.19 (0.08–0.43) | 0.33 (0.17–0.66) | 0.17 (0.07–0.43) | <0.01 |
| **Abbott (IgG N)** |  |  |  |  |  |  |
| S/C, median [min–max] | 0.06 [0.01–1.29] | 0.12 [0.01–0.22] | 0.36 [0.23–0.56] | 0.84 [0.57–1.31] | 2.55 [1.32–10.7] |  |
| Cases/Person-days (% ^a^) | 169/71901 (23.5) | 24/26150 (9.2) | 12/25121 (4.8) | 13/26620 (4.9) | 7/26205 (2.7) |  |
| Model 1 | 2.82 (1.84–4.33) | reference | 0.52 (0.26–1.03) | 0.52 (0.26–1.02) | 0.29 (0.12–0.67) | <0.01 |
| Model 2 | 2.91 (1.88–4.51) | reference | 0.53 (0.26–1.06) | 0.52 (0.27–1.03) | 0.29 (0.12–0.68) | <0.01 |
| Model 3 | 2.48 (1.59–3.88) | reference | 0.57 (0.29–1.15) | 0.59 (0.30–1.18) | 0.40 (0.17–0.94) | 0.07 |
| **Sysmex (IgG N)** |  |  |  |  |  |  |
| SU/mL, median [min–max] | 0.1 [0–8.4] | 2.6 [0–5.1] | 8.5 [5.2–13.6] | 21.7 [13.7–34.2] | 97.4 [34.8–94024] |  |
| Cases/Person-days (% ^a^) | 169/71901 (23.5) | 31/25107 (12.3) | 15/25944 (5.8) | 6/26395 (2.3) | 4/26650 (1.5) |  |
| Model 1 | 2.10 (1.43–3.09) | reference | 0.46 (0.25–0.86) | 0.18 (0.08–0.43) | 0.12 (0.04–0.35) | <0.01 |
| Model 2 | 2.14 (1.44–3.17) | reference | 0.45 (0.24–0.84) | 0.18 (0.08–0.44) | 0.12 (0.04–0.35) | <0.01 |
| Model 3 | 1.94 (1.30–2.91) | reference | 0.47 (0.25–0.87) | 0.19 (0.08–0.47) | 0.15 (0.05–0.42) | <0.01 |

Shown are the hazard ratios (95% confidence intervals).

Model 1 was adjusted for age (continuous) and sex (male or female).

Model 2 was additionally adjusted for job (doctors, nurses, allied health professionals, researchers, administrative staff, or others), occupational SARS-CoV-2 exposure risk (low, moderate, or high), body mass index (continuous), comorbid diseases (no or yes), immunosuppression (no or yes), use of tobacco products (no or yes), frequency of alcohol drinking (none, occasional, or weekly/daily drinker), number of household members (continuous), number of live-in school-aged children (0, 1, or ≥2), infection prevention score (continuous), spending ≥30 min in the 3Cs without mask (none, 1–5 times, or ≥6 times), and having dinner in a group of ≥5 people for >1 hours (none, 1–5 times, or ≥6 times).

Model 3 was further adjusted for anti-spike/RBD titers measured with the assay of the same company.

^a^ Incident rate per 10000 person-day

*Abbreviations*: 3Cs, crowded places, close-contact settings, and confined and enclosed spaces; COI, cut-off index; IgG, immunoglobulin G; N, nucleocapsid; Q, quartile; S/CO, signal to cut-off; SU, Sysmex unit.

**Supplemental Table 4.** Hazard ratios (95% confidence intervals) for subsequent SARS-CoV-2 infection across the baseline anti-nucleocapsid antibody index among participants, excluding non-regular staff (N=2,414)

| **Assays** | **Infection-naïve** | **Quartile of N antibodies among previously infected individuals** | | | | ***P* for trend** among previously infected individuals |
| --- | --- | --- | --- | --- | --- | --- |
|  |  | **Q1 (lowest)** | **Q2** | **Q3** | **Q4 (highest)** |  |
| **Roche (Total N)** |  |  |  |  |  |  |
| Cases/Person-days (% ^a^) | 168/ 67462 (24.9) | 34/23523 (14.5) | 11/24895 (4.4) | 12/25172 (4.8) | 5/25447 (2.0) |  |
| Model 1 | 1.83 (1.27–2.65) | reference | 0.29 (0.14–0.56) | 0.31 (0.16–0.61) | 0.13 (0.05–0.33) | <0.01 |
| Model 2 | 1.85 (1.26–2.71) | reference | 0.28 (0.14–0.56) | 0.31 (0.16–0.60) | 0.13 (0.05–0.34) | <0.01 |
| Model 3 | 1.50 (1.01–2.23) | reference | 0.29 (0.14–0.57) | 0.35 (0.18–0.67) | 0.16 (0.06–0.41) | <0.01 |
| **Abbott (IgG N)** |  |  |  |  |  |  |
| Cases/Person-days (% ^a^) | 168/ 67462 (24.9) | 26/24917 (10.4) | 16/23948 (6.7) | 13/24987 (5.2) | 7/25185 (2.8) |  |
| Model 1 | 2.61 (1.72–3.95) | reference | 0.64 (0.34–1.19) | 0.49 (0.25–0.95) | 0.26 (0.11–0.61) | <0.01 |
| Model 2 | 2.69 (1.76–4.11) | reference | 0.65 (0.35–1.22) | 0.49 (0.25–0.96) | 0.27 (0.12–0.62) | <0.01 |
| Model 3 | 2.30 (1.49–3.55) | reference | 0.70 (0.38–1.32) | 0.55 (0.28–1.08) | 0.36 (0.15–0.85) | 0.04 |
| **Sysmex (IgG N)** |  |  |  |  |  |  |
| Cases/Person-days (% ^a^) | 168/ 67462 (24.9) | 33/24084 (13.7) | 18/24466 (7.4) | 7/25006 (2.8) | 4/25481 (1.6) |  |
| Model 1 | 1.99 (1.37–2.89) | reference | 0.53 (0.30–0.94) | 0.20 (0.09–0.45) | 0.11 (0.04–0.32) | <0.01 |
| Model 2 | 2.01 (1.36–2.95) | reference | 0.51 (0.29–0.92) | 0.20 (0.09–0.45) | 0.12 (0.04–0.33) | <0.01 |
| Model 3 | 1.82 (1.22–2.70) | reference | 0.53 (0.30–0.95) | 0.21 (0.09–0.48) | 0.14 (0.05–0.40) | <0.01 |

This analysis done after excluding non-regular staff, including contractors, temporary staff, café staff, shop staff, and part-time registered medical doctors.

Shown are the hazard ratios (95% confidence intervals).

Model 1 was adjusted for age (continuous) and sex (male or female).

Model 2 was additionally adjusted for job (doctors, nurses, allied health professionals, researchers, administrative staff, or others), occupational SARS-CoV-2 exposure risk (low, moderate, or high), body mass index (continuous), comorbid diseases (no or yes), immunosuppression (no or yes), use of tobacco products (no or yes), frequency of alcohol drinking (none, occasional, or weekly/daily drinker), number of household members (continuous), number of live-in school-aged children (0, 1, or ≥2), infection prevention score (continuous), spending ≥30 min in the 3Cs without mask (none, 1–5 times, or ≥6 times), and having dinner in a group of ≥5 people for >1 hours (none, 1–5 times, or ≥6 times).

Model 3 was further adjusted for anti-spike/RBD titers measured with the assay of the same company.

^a^ Incident rate per 10000 person-day

*Abbreviations*: 3Cs, crowded places, close-contact settings, and confined and enclosed spaces; COI, cut-off index; IgG, immunoglobulin G; N, nucleocapsid; Q, quartile; S/CO, signal to cut-off; SU, Sysmex unit.
